# Supplementary material for: A conformational switch controlling the toxicity of the prion protein
Source: Nat Struct Mol Biol. 2022 Aug 10;29(8):831–40. doi: 10.1038/s41594-022-00814-7 (PMC9371974; doi:10.1038/s41594-022-00814-7)
Supplement: Supplementary file 1 — Supplementary Figures 1 and 2, Supplementary Table 1, Gene Block Sequences and Numerical Source Data for Supplementary Figure 2C [file 41594_2022_814_MOESM1_ESM.pdf]

nature portfolio

https://doi.org/10.1038/s41594-022-00814-7

Supplementary information

A conformational switch controlling the toxicity of the prion protein

In the format provided by the authors and unedited

Supplementary Figure 1

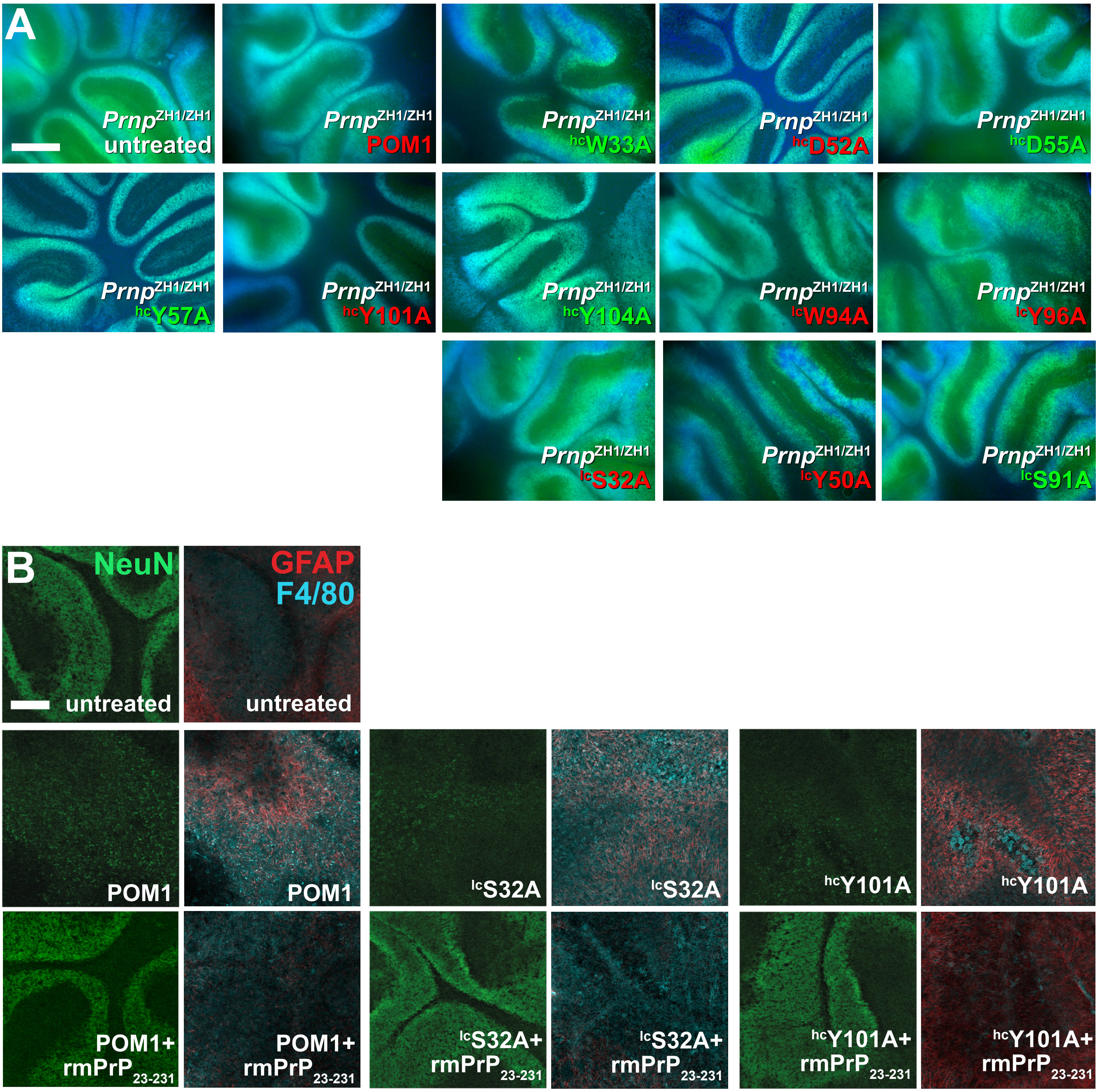

Supplementary Figure 2

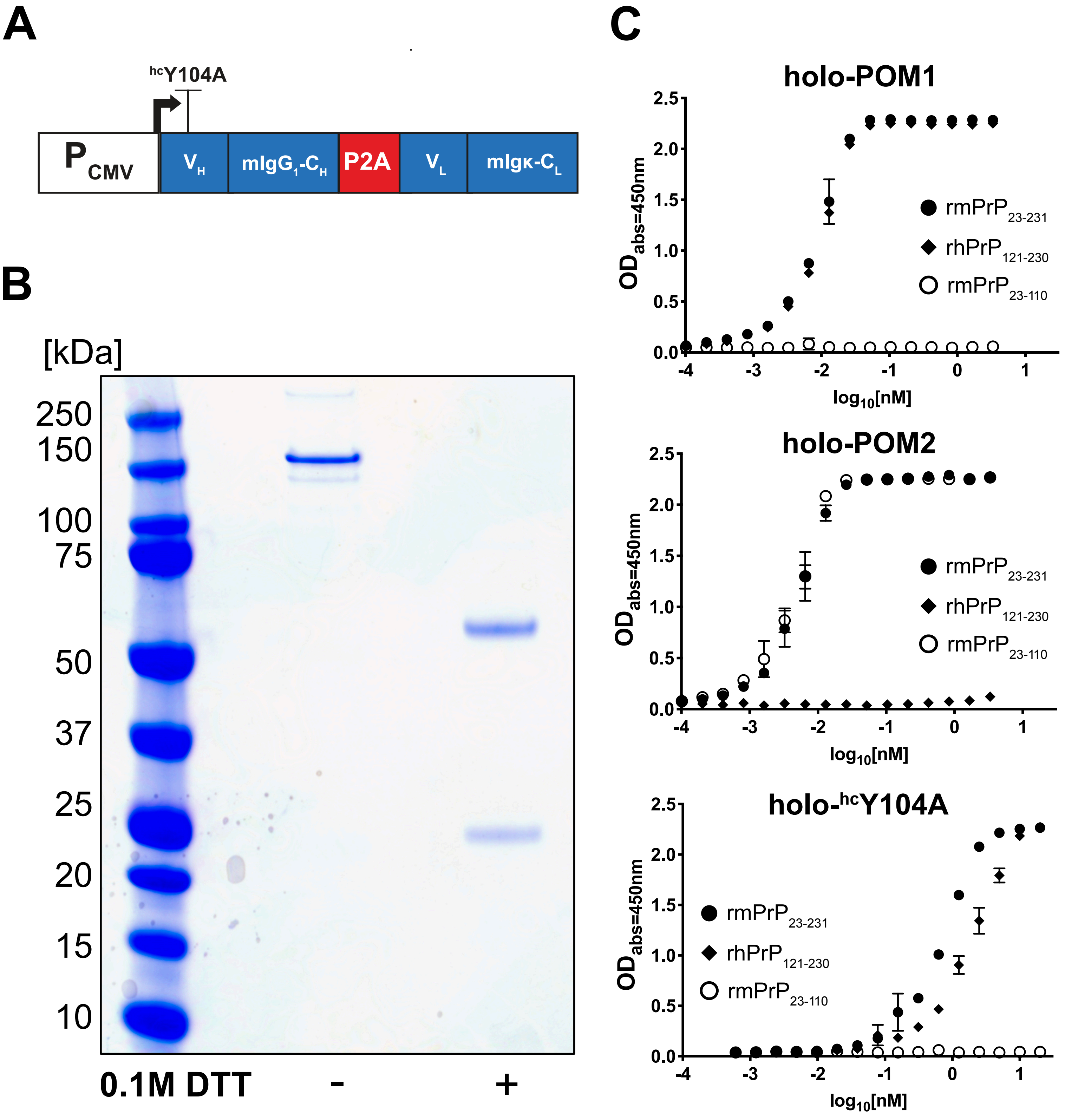

***Supplementary Figure 1*. (A)** Representative fluorescent micrographs of *Prnp*$^{ZH1/ZH1}$ COCS treated with pomologs and morphometric quantification. In all cases, neuronal viability is similar to that of controls, indicating that neither POM1 nor any of the pomologs exert a toxic effect onto PrP-deficient tissue. Innocuous / protective pomologs are colored green, toxic pomologs red. Scale bar = 500 µm. Quantification of all biological replicates is given in Extended Data Figure 8a. **(B)** Immunofluorescent stainings of COCS with NeuN (neurons), GFAP (astrocytes) and F-4/80 (microglia and macrophages). The high-affinity toxic pomologs ^{lc}S32A and ^{hc}Y101A were administered to *tga20* COCS, either alone or after preincubation with equimolar amounts of soluble rmPrP$_{23\text{-}231}$. POM1, ^{lc}S32A and ^{lc}Y101A induced profound neuronal loss, astrogliosis and activation of microglia, which was completely prevented by pre-incubation with their cognate antigen. Scale bar = 200 µm. Quantification of all biological replicates is given in Extended Data Figure 8c.

***Supplementary Figure* 2**. **(A)** Schematic depiction of the vector for recombinant mammalian expression of holo-^{hc}Y104A. A bicistronic expression cassette encoding the variable domains of ^{hc}Y104A were grafted onto a murine IgG1 and Igκ backbone driven by a cytomegalovirus promoter. A P2A self-cleaving site at the 3' end of the heavy-chain fragment results in co-translational cleavage of the polyprotein. **(B)** SDS-PAGE of purified holo-^{hc}Y104A in the presence and absence of 0.1M Dithiothreitol (DTT). **(C)** Indirect ELISA of holo-^{hc}Y104A and holo-POM1 showing (sub-) nanomolar affinity to full-length, recombinant mouse PrP (mPrP$_{23\text{-}230}$) and a globular domain fragment of recombinant human PrP (mPrP$_{121\text{-}231}$). As a control, holo-wtPOM2, which targets the flexible tail, bound to both rmPrP$_{23\text{-}231}$ and rmPrP$_{23\text{-}110}$, but not to rhPrP$_{121\text{-}230}$. Neither holo-^{hc}Y104A nor holo-POM1 bind to rmPrP$_{23\text{-}110}$. Nanomolar antibody concentrations are plotted in $\log_{10}$ scales on the abscissa. Data points are given as means, bars indicate standard deviation.

Table 1A

Res N*	chain	ΔG(complex)	
32	L	0.44	POM1 residues
50	L	1.96	
91	L	2.09	
92	L	0.27	
93	L	0.28	
94	L	1.39	
96	L	0.35	
33	H	2.88	
52	H	2.47	
54	H	-0.02	
55	H	1.37	
57	H	1.76	
59	H	-0.01	
101	H	0.59	
103	H	-0.03	
104	H	4.91	
139	A	-0.01	PrP residues
146	A	-0.01	
138	A	0.11	
143	A	-0.21	
145	A	0.31	
141	A	0.39	
212	A	0.78	
140	A	0.93	
147	A	1.9	
144	A	3.44	
208	A	3.86	

Table 1B

	k_a(1/Ms)	k_d(1/s)	K_D(nM)	
POM1	6.4 x 10^4	3.1 x 10^-4	4.8	
hcW33A	no binding	no binding	no binding	CDR_H1 loop
hcD52A	3.3 x 10^5	4.6 x 10^-2	103	CDR_H2 loop
hcD55A	1 x 10^5	3.7 x 10^-2	372	
hcY57A	7.7 x 10^4	3.1 x 10^-2	406	
hcY101A	6.3 x 10^4	7.1 x 10^-4	11	CDR_H3 loop
hcY104A	3.6 x 10^4	2.1 x 10^-4	8.8	
lcS32A	6.4 x 10^4	5.9 x 10^-4	10	CDR_L1 loop
lcY50A	5.7 x 10^4	8.2 x 10^-3	313	CDR_L2 loop
lcS91A	1.3 x 10^4	1.8 x 10^-2	1430	CDR_L3 loop
lcW94A	3.1 x 10^5	2.7 x 10^-2	201	
lcY96A	6.2 x 10^4	7.4 x 10^-3	123	

Gene Block Sequences Page 1/2

hcY104A gene block

GCCACCATGTATCGAATGCAACTGCTGTCATGTATTGCCCTCAGCCTGGCACTGGTGACAAACTCCCAAGTCCAGCTTCAGCAATCAGGAACTG
AGCTTGTTATGCCGGGTGCGTCAGTAAAAATGTCCTGCAAGGCTTCTGGCTACACTTTCACTGATTATTGGATGCACTGGGTCAAGCAGAGAC
CTGGGCAGGGGCTCGAGTGGATCGGCAGTATTGACCCATCAGACTCAtatACGTCTCACAACGAGAAATTTAAAGGAAAAGCTACTTTGACAG
TTGACGAAAGCTCAAGCACGGCGTACATGCAGCTCTCATCCTTGACCTCTGAGGATTCCGCAGTTTACTTTTGCTCAAGAAGCGGCTATGGTTA
TgccGCTATGGAATATTGGGGGCAAGGAACGTCCGTCACCGTATCCTCAGCCAAGACCACCCCCCCTCAGTGTACCCGCTGGCGCCGGGGAGT
GCTGCGCAAACGAACTCAATGGTGACGCTTGGTTGCTTGGTGAAAGGGTACTTCCCTGAGCCAGTTACGGTCACATGGAATTCTGGCTCTTTG
TCATCTGGCGTGCATACATTCCCCGCCGTGCTGCAGAGCGACCTGTACACGCTGAGCAGTTCAGTCACTGTGCCAAGTTCCACATGGCCCTCCGA
GACTGTCACCTGTAATGTGGCTCACCCCGCCTCTAGTACTAAGGTAGACAAGAAAATAGTCCCGCGGGATTGCGGTTGTAAGCCATGCATTTG
TACCGTACCCGAAGTTAGCTCAGTCTTTATATTTCCGCCTAAGCCGAAAGACGTGCTGACAATCACCCTGACCCCCAAGGTAACTTGTGTTGTTG
TGGACATTTCCAAGGACGATCCCGAAGTTCAGTTCTCTTGGTTTGTTGACGACGTCGAGGTTCATACCGCACAAACGCAACCAAGGGAGGAAC
AATTCAACTCAACATTTCGCTCAGTGTCCGAGCTGCCGATAATGCATCAGGATTGGCTTAATGGAAAGGAGTTCAAGTGTCGGGTCAATTCCG
CAGCGTTTCCTGCGCCCATTGAGAAAACTATTTCCAAGACAAAAGGACGCCCAAAGGCACCCCAAGTCTACACTATACCCCCACCCAAAGAGCAG
ATGGCGAAGGACAAGGTTTCCCTCACTTGCATGATCACAGACTTCTTTCCTGAAGACATCACCGTAGAGTGGCAGTGGAATGGCCAGCCGGCC
GAAAACTACAAAAACACCCAGCCTATCATGGATACCGATGGAAGCTACTTCGTCTATAGCAAGTTGAACGTGCAAAAATCCAATTGGGAAGCC
GGAAATACATTCACGTGCTCCGTGTTGCACGAAGGCCTTCACAATCATCATACAGAGAAGTCACTCTCACATAGCCCTGGTAAGGGAAGTGGA
GCTACAAACTTCTCCCTCCTTAAGCAAGCCGGCGATGTGGAAGAAAACCCAGGGCCGATGTATCGCATGCAGCTTCTGTCATGCATTGCTCTGT
CATTGGCACTGGTTACTAACTCAGACATAGTACTCACACAATCTCCAGCGATTCTGAGCGTTTCTCCTGGGGAAAGAGTATCCTTCTCTTGCCGA
GCTAGTCAGAACATTGGGACCTCAATTCATTGGTATCAACAGAGAACGAACGAGAGTCCCAGGCTCATTATCAAGTATGCATCAGAATCAATTT
CAGGGATACCCTCCAGATTCAGCGGCTCCGGGTCTGGTACGGATTTTACACTTAGCATAAATTCAGTGGAAAGCGAGGACATAGCAGACTACT
ATTGTCAGCAAAGTAATACCTGGCCATACACATTTGGTGGGGGGACGAAGTTGGAACTGAAACGGGCGGATGCCGCTCCCACGGTAAGTATC
TTCCCCCCGTCTTCAGAGCAACTTACCAGCGGCGGAGCATCCGTAGTCTGCTTCTTGAATAACTTTTATCCAAAGGACATTAATGTTAAATGGAA
AATAGATGGTTCAGAAAGACAGAACGGGGTTCTTAACTCATGGACCGATCAGGATAGCAAAGACAGCACGTATAGCATGAGCTCAACCCTGA
CCCTCACGAAGGATGAATACGAGCGCCATAACTCCTACACTTGTGAAGCCACACACAAAACTAGTACTTCACCGATAGTAAAGTCTTTTAACCGG
AACGAGTGTTAG

AAV insert: mNeonGreen-P2A-PrP^{C}

ATGGTGAGCAAGGGCGAGGAGGATAACATGGCCTCTCTCCCAGCGACACATGAGTTACACATCTTTGGCTCCATCAACGGTGTGGACTTTGAC
ATGGTGGGTCAGGGCACCGGCAATCCAAATGATGGTTATGAGGAGTTAAACCTGAAGTCCACCAAGGGTGACCTCCAGTTCTCCCCCTGGATT
CTGGTCCCTCATATCGGGTATGGCTTCCATCAGTACCTGCCCTACCCTGACGGGATGTCGCCTTTCCAGGCCGCCATGGTAGATGGCTCCGGAT
ACCAAGTCCATCGCACAATGCAGTTTGAAGATGGTGCCTCCCTTACTGTTAACTACCGCTACACCTACGAGGGAAGCCACATCAAAGGAGAGGC
CCAGGTGAAGGGGACTGGTTTCCCTGCTGACGGTCCTGTGATGACCAACTCGCTGACCGCTGCGGACTGGTGCAGGTCGAAGAAGACTTACC
CCAACGACAAAACCATCATCAGTACCTTTAAGTGGAGTTACACCACTGGAAATGGCAAGCGCTACCGGAGCACTGCGCGGACCACCTACACCTT
TGCCAAGCCAATGGCGGCTAACTATCTGAAGAACCAGCCGATGTACGTGTTCCGTAAGACGGAGCTCAAGCACTCCAAGACCGAGCTCAACTT
CAAGGAGTGGCAAAAGGCCTTTACCGATGTGATGGGCATGGACGAGCTGTACAAGGGCAGCGGCGCCACGAACTTCTCTCTGTTAAAGCAA
GCAGGCGACGTGGAAGAAAACCCCGGTCCCATGGCGAACCTTGGCTACTGGCTGCTGGCCCTCTTTGTGACTATGTGGACTGATGTCGGCCTC
TGCAAAAAGCGGCCAAAGCCTGGAGGGTGGAACACCGGTGGAAGCCGGTATCCCGGGCAGGGAAGCCCTGGAGGCAACCGTTACCCACCTC
AGGGTGGCACCTGGGGGCAGCCCCACGGTGGTGGCTGGGGACAACCCCATGGGGGCAGCTGGGGACAACCTCATGGTGGTAGTTGGGGTC
AGCCCCATGGCGGTGGATGGGGCCAAGGAGGGGGTACCCATAATCAGTGGAACAAGCCCAGCAAACCAAAAACCAACCTCAAGCATGTGGC
AGGGGCTGCGGCAGCTGGGGCAGTAGTGGGGGGCCTTGGTGGCTACATGCTGGGGAGCGCCATGAGCAGGCCCATGATCCATTTTGGCAA
CGACTGGGAGGACCGCTACTACCGTGAAAACATGTACCGCTACCCTAACCAAGTGTACTACAGGCCAGTGGATCAGTACAGCAACCAGAACAA
CTTCGTGCACGACTGCGTCAATATCACCATCAAGCAGCACACGGTCACCACCACCACCAAGGGGGAGAACTTCACCGAGACCGATGTGAAGAT
GATGGAGCGCGTGGTGGAGCAGATGTGCGTCACCCAGTACCAGAAGGAGTCCCAGGCCTATTACGACGGGAGAAGATCCAGCAGCACCGTG
CTTTTCTCCTCCCCTCCTGTCATCCTCCTCATCTCCTTCCTCATCTTCCTGATCGTGGGATGA

Gene Block Sequences Page 2/2

R207A mutation in AAV insert: mNeonGreen-P2A-PrP

ATGGTGAGCAAGGGCGAGGAGGATAACATGGCCTCTCTCCCAGCGACACATGAGTTACACATCTTTGGCTCCATCAACGGTGTGGACTTTGAC
ATGGTGGGTCAGGGCACCGGCAATCCAAATGATGGTTATGAGGAGTTAAACCTGAAGTCCACCAAGGGTGACCTCCAGTTCTCCCCCTGGATT
CTGGTCCCTCATATCGGGTATGGCTTCCATCAGTACCTGCCCTACCCTGACGGGATGTCGCCTTTCCAGGCCGCCATGGTAGATGGCTCCGGAT
ACCAAGTCCATCGCACAATGCAGTTTGAAGATGGTGCCTCCCTTACTGTTAACTACCGCTACACCTACGAGGGAAGCCACATCAAAGGAGAGGC
CCAGGTGAAGGGGACTGGTTTCCCTGCTGACGGTCCTGTGATGACCAACTCGCTGACCGCTGCGGACTGGTGCAGGTCGAAGAAGACTTACC
CCAACGACAAAACCATCATCAGTACCTTTAAGTGGAGTTACACCACTGGAAATGGCAAGCGCTACCGGAGCACTGCGCGGACCACCTACACCTT
TGCCAAGCCAATGGCGGCTAACTATCTGAAGAACCAGCCGATGTACGTGTTCCGTAAGACGGAGCTCAAGCACTCCAAGACCGAGCTCAACTT
CAAGGAGTGGCAAAAGGCCTTTACCGATGTGATGGGCATGGACGAGCTGTACAAGGGCTCTGGCGCTACAAACTTTAGTCTTTTGAAACAAG
CAGGGGACGTGGAAGAGAACCCAGGACCCATGGCCAATTTGGGCTACTGGCTGCTCGCTCTCTTCGTCACAATGTGGACTGACGTCGGGCTCT
GTAAGAAAAGGCCCAAGCCCGGAGGATGGAATACAGGTGGGTCTCGATACCCAGGGCAGGGCAGTCCTGGGGGGAATAGATACCCTCCACA
GGGCGGGACCTGGGGTCAACCTCATGGTGGGGGTTGGGGTCAACCACATGGCGGCAGTTGGGGCCAACCTCACGGAGGCTCCTGGGGGCA
GCCCCACGGAGGAGGATGGGGTCAGGGAGGCGGCACCCATAATCAGTGGAACAAACCAAGCAAGCCTAAGACTAACTTGAAACATGTAGCA
GGAGCTGCCGCAGCCGGGGCAGTCGTTGGCGGGTTGGGGGGGTACATGCTTGGAAGCGCAATGAGTCGCCCCATGATCCATTTTGGCAATG
ACTGGGAGGATCGCTATTACCGAGAGAATATGTATAGATATCCCAACCAAGTATATTACCGCCCTGTTGACCAGTATTCAAACCAAAACAATTT
CGTCCATGATTGTGTGAATATAACAATAAAACAGCATACCGTGACAACCACCACTAAAGGCGAAAATTTCACCGAAACAGATGTAAAAATGAT
GGAGGCAGTCGTCGAGCAAATGTGCGTTACCCAGTACCAAAAGGAAAGCCAAGCCTATTACGATGGACGCCGGTCTAGTTCAACTGTACTCTT
TTCAAGCCCCCCAGTTATATTGCTCATCTCTTTTCTGATATTTCTCATTGTAGGGTAA

R207C/I138C mutation in AAV insert: mNeonGreen-P2A-PrP

ATGGTGAGCAAGGGCGAGGAGGATAACATGGCCTCTCTCCCAGCGACACATGAGTTACACATCTTTGGCTCCATCAACGGTGTGGACTTTGAC
ATGGTGGGTCAGGGCACCGGCAATCCAAATGATGGTTATGAGGAGTTAAACCTGAAGTCCACCAAGGGTGACCTCCAGTTCTCCCCCTGGATT
CTGGTCCCTCATATCGGGTATGGCTTCCATCAGTACCTGCCCTACCCTGACGGGATGTCGCCTTTCCAGGCCGCCATGGTAGATGGCTCCGGAT
ACCAAGTCCATCGCACAATGCAGTTTGAAGATGGTGCCTCCCTTACTGTTAACTACCGCTACACCTACGAGGGAAGCCACATCAAAGGAGAGGC
CCAGGTGAAGGGGACTGGTTTCCCTGCTGACGGTCCTGTGATGACCAACTCGCTGACCGCTGCGGACTGGTGCAGGTCGAAGAAGACTTACC
CCAACGACAAAACCATCATCAGTACCTTTAAGTGGAGTTACACCACTGGAAATGGCAAGCGCTACCGGAGCACTGCGCGGACCACCTACACCTT
TGCCAAGCCAATGGCGGCTAACTATCTGAAGAACCAGCCGATGTACGTGTTCCGTAAGACGGAGCTCAAGCACTCCAAGACCGAGCTCAACTT
CAAGGAGTGGCAAAAGGCCTTTACCGATGTGATGGGCATGGACGAGCTGTACAAGGGCTCCGGTGCCACTAATTTTAGCTTGCTGAAGCAG
GCCGGGGACGTGGAAGAGAACCCTGGCCCTATGGCTAACCTTGGTTACTGGCTGCTTGCTCTCTTTGTTACTATGTGGACCGATGTCGGCCTG
TGCAAAAAGCGACCAAAGCCAGGTGGGTGGAATACAGGCGGGTCCAGATACCCAGGGCAGGGAAGCCCAGGGGGAAATAGGTATCCCCCT
CAAGGTGGTACTTGGGGTCAACCACACGGAGGCGGCTGGGGTCAACCCCATGGAGGCTCTTGGGGGCAACCTCATGGTGGCAGTTGGGGTC
AACCTCATGGAGGCGGTTGGGGCCAAGGAGGAGGCACTCACAACCAGTGGAATAAACCATCCAAACCCAAAACCAACCTTAAACACGTTGCA
GGCGCTGCCGCAGCAGGGGCCGTTGTAGGCGGACTCGGGGGCTATATGCTTGGCTCTGCCATGAGTCGGCCCATGTGTCATTTCGGCAATGA
TTGGGAGGATAGGTATTATCGGGAGAATATGTACCGATATCCAAATCAAGTATACTACAGACCAGTAGACCAATACTCAAATCAAAACAATTT
CGTTCACGACTGTGTGAACATAACCATCAAGCAACATACAGTGACTACAACAACCAAAGGGGAAAATTTTACTGAGACCGACGTAAAAATGAT
GGAGTGTGTTGTTGAACAAATGTGTGTAACCCAGTACCAAAAAGAGAGTCAGGCTTACTACGATGGCAGACGGTCATCCAGTACAGTGCTTT
TTTCTTCCCCACCCGTAATACTCCTGATCTCCTTTCTGATTTTCTTGATAGTAGGCTGA

Numerical Source Data Suppl. Fig. 2C

holo-Y104A

$\log_{10}$[nM]	$mPrP_{23-230}$		$mPrP_{121-230}$		$mPrP_{23-110}$	
1.301029996	2.268	2.265	2.277	2.27	0.044	0.044
1	2.254	2.253	2.164	2.206	0.043	0.043
0.698970004	2.218	2.214	1.743	1.843	0.035	0.041
0.397940009	2.087	2.068	1.251	1.435	0.042	0.044
0.096910013	1.598	1.599	0.842	0.967	0.036	0.041
-0.204119983	1.004	1.015	0.445	0.489	0.077	0.044
-0.505149978	0.585	0.566	0.254	0.325	0.041	0.042
-0.806179974	0.567	0.306	0.176	0.192	0.04	0.036
-1.10720997	0.184	0.172	0.281	0.137	0.04	0.035
-1.408239965	0.116	0.101	0.073	0.077	0.044	0.043
-1.709269961	0.076	0.072	0.059	0.063	0.053	0.042
-2.010299957	0.057	0.051	0.047	0.05	0.044	0.04
-2.311329952	0.052	0.041	0.047	0.049	0.046	0.041
-2.612359948	0.048	0.042	0.044	0.046	0.05	0.044
-2.913389944	0.042	0.041	0.042	0.039	0.042	0.038
-3.214419939	0.043	0.041	0.043	0.051	0.039	0.037

holo-POM2

$\log_{10}$[nM]	$mPrP_{23-230}$		$mPrP_{121-230}$		$mPrP_{23-110}$	
0.522878745	2.273	2.27	0.125	0.119	2.269	2.266
0.22184875	2.26	2.252	0.078	0.09	2.251	2.249
-0.079181246	2.309	2.273	0.071	0.08	2.259	2.246
-0.380211242	2.276	2.277	0.054	0.069	2.265	2.245
-0.681241237	2.27	2.258	0.047	0.052	2.261	2.248
-0.982271233	2.256	2.253	0.042	0.049	2.252	2.246
-1.283301229	2.24	2.242	0.038	0.036	2.248	2.244
-1.584331224	2.2	2.192	0.045	0.048	2.241	2.24
-1.88536122	1.864	1.972	0.052	0.039	2.053	2.115
-2.186391216	1.213	1.375	0.054	0.042	1.13	1.469
-2.487421211	0.662	0.913	0.067	0.044	0.785	0.952
-2.788451207	0.345	0.364	0.038	0.036	0.365	0.616
-3.089481203	0.214	0.229	0.077	0.041	0.266	0.298
-3.390511198	0.126	0.138	0.042	0.042	0.122	0.176
-3.691541194	0.106	0.084	0.052	0.05	0.119	0.114
-3.99257119	0.064	0.069	0.095	0.11	0.063	0.092

holo-POM1

$\log_{10}$[nM]	$mPrP_{23-230}$		$mPrP_{121-230}$		$mPrP_{23-110}$	
0.522878745	2.305	2.261	2.251		0.058	0.058
0.22184875	2.314	2.26	2.24		0.055	0.052
-0.079181246	2.313	2.252	2.24		0.041	0.051
-0.380211242	2.31	2.252	2.24		0.052	0.052
-0.681241237	2.311	2.251	2.251		0.05	0.053
-0.982271233	2.323	2.258	2.251		0.049	0.055
-1.283301229	2.319	2.247	2.231		0.048	0.048
-1.584331224	2.132	2.065	2.043		0.044	0.045
-1.88536122	1.638	1.328	1.373		0.054	0.05
-2.186391216	0.89	0.863	0.781		0.123	0.044
-2.487421211	0.507	0.497	0.451		0.048	0.046
-2.788451207	0.27	0.253	0.247		0.047	0.044
-3.089481203	0.181	0.177	0.182		0.05	0.049
-3.390511198	0.138	0.117	0.109		0.048	0.046
-3.691541194	0.122	0.077	0.082		0.051	0.047
-3.99257119	0.074	0.063	0.061		0.05	0.052
