# Supplementary material for: A conformational switch controlling the toxicity of the prion protein
Source: Nat Struct Mol Biol. 2022 Aug 10;29(8):831–40. doi: 10.1038/s41594-022-00814-7 (PMC9371974; doi:10.1038/s41594-022-00814-7)
Supplement: Supplementary file 2 — Reporting Summary [file 41594_2022_814_MOESM2_ESM.pdf]

nature portfolio

Corresponding author(s): Karl Frontzek
Luca Varani
Adriano Aguzzi

Last updated by author(s): May 26, 2022

# Reporting Summary

Nature Portfolio wishes to improve the reproducibility of the work that we publish. This form provides structure for consistency and transparency in reporting. For further information on Nature Portfolio policies, see our Editorial Policies and the Editorial Policy Checklist.

## Statistics

For all statistical analyses, confirm that the following items are present in the figure legend, table legend, main text, or Methods section.

| n/a | Confirmed | |
|---|---|---|
| ☐ | ☒ | The exact sample size (*n*) for each experimental group/condition, given as a discrete number and unit of measurement |
| ☐ | ☒ | A statement on whether measurements were taken from distinct samples or whether the same sample was measured repeatedly |
| ☐ | ☒ | The statistical test(s) used AND whether they are one- or two-sided<br>*Only common tests should be described solely by name; describe more complex techniques in the Methods section.* |
| ☐ | ☒ | A description of all covariates tested |
| ☐ | ☒ | A description of any assumptions or corrections, such as tests of normality and adjustment for multiple comparisons |
| ☐ | ☒ | A full description of the statistical parameters including central tendency (e.g. means) or other basic estimates (e.g. regression coefficient) AND variation (e.g. standard deviation) or associated estimates of uncertainty (e.g. confidence intervals) |
| ☐ | ☒ | For null hypothesis testing, the test statistic (e.g. $F$, $t$, $r$) with confidence intervals, effect sizes, degrees of freedom and $P$ value noted<br>*Give P values as exact values whenever suitable.* |
| ☒ | ☐ | For Bayesian analysis, information on the choice of priors and Markov chain Monte Carlo settings |
| ☒ | ☐ | For hierarchical and complex designs, identification of the appropriate level for tests and full reporting of outcomes |
| ☐ | ☒ | Estimates of effect sizes (e.g. Cohen's *d*, Pearson's *r*), indicating how they were calculated |

*Our web collection on statistics for biologists contains articles on many of the points above.*

## Software and code

Policy information about availability of computer code

| | |
|---|---|
| Data collection | We used RStudio (1.1.383) with R (3.4.2), CDApps (4.0), FlowJo (10) |
| Data analysis | Custom code was used to analyze Allen Mouse Brain Atlas data, which can be found in the supplementary data. |

For manuscripts utilizing custom algorithms or software that are central to the research but not yet described in published literature, software must be made available to editors and reviewers. We strongly encourage code deposition in a community repository (e.g. GitHub). See the Nature Portfolio guidelines for submitting code & software for further information.

## Data

Policy information about availability of data

All manuscripts must include a data availability statement. This statement should provide the following information, where applicable:

- Accession codes, unique identifiers, or web links for publicly available datasets
- A description of any restrictions on data availability
- For clinical datasets or third party data, please ensure that the statement adheres to our policy

All source data, e.g. numeric source data, uncropped western blot gels including annotation thereofs as well as unique DNA sequences, is available in the manuscript or the supplemen-tary materials. The following, publicly available data was used: Allen Mouse Brain Atlas, entries 71717640 and 227540 (https://mouse.brain-map.org); Biological Magnetic Resonance Data Bank, entry 16071 (https://bmrb.io/); RCSB Protein Data Bank, entries 1XYX and 4H88 (https://www.rcsb.org). Additionally, all unique biological materials used in the manuscript are readily available from the authors.

## Human research participants

Policy information about studies involving human research participants and Sex and Gender in Research.

| | |
|---|---|
| Reporting on sex and gender | *Use the terms sex (biological attribute) and gender (shaped by social and cultural circumstances) carefully in order to avoid confusing both terms. Indicate if findings apply to only one sex or gender; describe whether sex and gender were considered in study design whether sex and/or gender was determined based on self-reporting or assigned and methods used. Provide in the source data disaggregated sex and gender data where this information has been collected, and consent has been obtained for sharing of individual-level data; provide overall numbers in this Reporting Summary. Please state if this information has not been collected. Report sex- and gender-based analyses where performed, justify reasons for lack of sex- and gender-based analysis.* |
| Population characteristics | *Describe the covariate-relevant population characteristics of the human research participants (e.g. age, genotypic information, past and current diagnosis and treatment categories). If you filled out the behavioural & social sciences study design questions and have nothing to add here, write "See above."* |
| Recruitment | *Describe how participants were recruited. Outline any potential self-selection bias or other biases that may be present and how these are likely to impact results.* |
| Ethics oversight | *Identify the organization(s) that approved the study protocol.* |

Note that full information on the approval of the study protocol must also be provided in the manuscript.

# Field-specific reporting

Please select the one below that is the best fit for your research. If you are not sure, read the appropriate sections before making your selection.

☒ Life sciences ☐ Behavioural & social sciences ☐ Ecological, evolutionary & environmental sciences

For a reference copy of the document with all sections, see nature.com/documents/nr-reporting-summary-flat.pdf

# Life sciences study design

All studies must disclose on these points even when the disclosure is negative.

| | |
|---|---|
| Sample size | No statistical methods were used to predetermine sample size, moreover, sample size was chosen based on previously performed experiments, as published before (PMID 26821311, 25710374, 23903654, 23133383) |
| Data exclusions | ED Fig 6C: immunohistochemical analysis of slice cultures is known to show large variability (Falsig et al., PLOS Pathogens 2012, PMID 23133383) , here, one extreme outlier was excluded from the analysis (y=2046.3%, p<0.05, extreme studentized deviate method). |
| Replication | Biological replicates from in vitro experiments correspond to different culture passages. Biological replicates from organtypic slice culture correspond to slices from different animals. Experiments depicted in Figures 2D, 3D-F, 4A, 4B, 4D+F, 7A, ED 3E, ED 6A,B,G,I were successfully replicated in two independent runs. Experiments depicted in Figures 1C,D,F, 2B+C, 3B+C, 6D+E, ED1, ED4, ED5D were successfully replicated in three or more independent runs. Experiments depicted in the following images contain multiple, independent, biological replicates, but were only performed once: 2E, 2F, 3A, 4C, 4E, 4G, 5A, ED 3B, ED 6B-F, ED 6H, ED7B+C. Experiments depicted in ED 3C+D were performed once with two independent, biological replicates. POM1 and POM19 of experiment from Fig ED 2D were replicated successfully, we did not attempt replication of POM5 and POM8, because it was later orthogonal verified in MDS and NMR.<br>Due to disproportionate effort and in agreement with standard practice, intracerebral/-venous injections and in vivo imaging of animals (depicted in Fig. 6, ED 7+8, although they were performed with several independent, biological replicates), NMR and SRCD analyses (Fig. 3, 5, 7D+E) as well as antibody phage display (Fig. ED 9) were only performed once. |
| Randomization | Animals were randomly assigned to treatment groups. Brain slices were pooled from different animals and randomly selected for treatment. Similarly, allocation of cells in in vitro experiments was random.<br>Sections used for imaging were selected randomly and were analyzed equally with no sub-sampling thus omitting a need for randomization. |
| Blinding | NeuN morphometry was performed blindly. Prion disease symptoms of AAV-treated mice were assessed by an animal caretaker blinded to the treatment (P.S.). In the case of other experiments, blinding was not attempted, in parts because testing conditions were evident from the experimental data. However, quantifications were performed using computational pipelines applied equally to all conditions and replicates for a given experiment. |

# Reporting for specific materials, systems and methods

We require information from authors about some types of materials, experimental systems and methods used in many studies. Here, indicate whether each material, system or method listed is relevant to your study. If you are not sure if a list item applies to your research, read the appropriate section before selecting a response.

## Materials & experimental systems

| n/a | Involved in the study |
|---|---|
| ☐ | ☒ Antibodies |
| ☐ | ☒ Eukaryotic cell lines |
| ☒ | ☐ Palaeontology and archaeology |
| ☐ | ☒ Animals and other organisms |
| ☒ | ☐ Clinical data |
| ☒ | ☐ Dual use research of concern |

## Methods

| n/a | Involved in the study |
|---|---|
| ☒ | ☐ ChIP-seq |
| ☐ | ☒ Flow cytometry |
| ☐ | ☒ MRI-based neuroimaging |

# Antibodies

Antibodies used

Please specify the original source (commercial or other. If they are commercially available, please provide information on their supplier name, catalog number, clone name and lot number, as applicable) for all the other POM whole IgG antibodies, here in the reporting summary.

Please ensure all the antibodies referenced in the manuscript are listed here in the reporting summary (with supplier name, catalog number, clone name and lot number, as applicable). For example: Alexa Fluor 594 Rabbit Anti-Goat (IgG) secondary antibody

Please provide the catalog number, and lot number, as applicable, for all the following antibodies listed, here in the reporting summary. For example: phospho-eIF2α (Cell Signaling Technologies), etc.

POM1 and POM2 whole IgG antibodies are available via Merck (POM1 MABN2285, POM2 MABN2298).
Other POM whole IgG antibodies are originially derived by immunization of Prnp knock-out mice and subsequent monoclonal expansion of hybridomas as laid out in Polymenidou et al., PLOS ONE 2008, PMID 19060956. Generation of Fab and scFv fragments was performed using pepsin digestion of holo-IgGs and periplasmic expression, respectively, as reported extensively in Sonati et al., Nature 2013, PMID 23903654. Generation procedures of POM1 holo-IgG and scFv mutants by eukaroyotic and periplasmic expression, respectively, is listed in the materials and methods section of the present manuscript, the antibodies can be obtained through the authors.

Antibody sequences are deposited on NCBI (POM1 heavy chain 4DGI_H, POM1 light chain 4DGI_L, POM2 heavy chain 4J8R_D, POM2 light chain 4J8R_C) and in Polymenidou et al., PLOS One 2008, or are described in Materials and Methods (POM1 mutants) or can be obtained from the authors (Fab fragments displayed in Fig 7A).

Other antibodies used are as follows, given as Name, Catalog #, Company
anti-Fd, PC075, Binding Site
anti-Myc-tag , AB9106, Abcam
anti-monomeric NeonGreen, 32F6-100, Chromotek
anti-phospho-eIF2α, 3398, Cell Signaling Technologies
anti-eIF2α, 5324, Cell Signaling Technologies
anti-Actin Antibody, MAB1501R, Millipore
anti-GFAP, Z0334, DAKO/Agilent
anti-F4/80, MCA497G, Serotec/BioRad
anti-human F(ab')2-alkaline phosphatase conjugated antibody, SAB3701239, Sigma
Streptavidin/HRP, 554066, BD Biosciences
anti-mouse IgG, 115-035-062, Jackson ImmunoResearch
anti-rabbit IgG, 111-035-045, Jackson ImmunoResearch
Alexa594-conjugated goat anti-rabbit IgG , A-11012, Thermo Fisher
Alexa594-conjugated rabbit anti-goat IgG , A-11037, Thermo Fisher
Alexa647-conjugated goat anti-rat IgG , A-21247, Thermo Fisher
Alexa488-conjugated goat anti-mouse IgG, A-28175, Thermo Fisher
anti-NeuN Antibody,Alexa Fluor®488 conjugated, MAB377X, Merck

Validation

Validation information of commercial antibodies is extracted from manufacturer's websites and listed in the format (abbrevations are explained at the bottom of this field):
Target/Name, 1°/2°/conjugated 1°+2° AB, Host, Reactivity, Applications-Manufacturer, Application-Manuscript, Catalog # , Clone (if applicable), Dilution used in the manuscript, Company, Citation PMID, Application in paper different from manufacturer's suggestion (Yes/No), Citation contains validation of application from paper (Yes/No)

anti-Fd, 1, S, H, Immunoelectrophoresis (IEP), Radial Immunodiffusion (RID), ELISA, PC075, n/a, 1:1000, Binding Site, 32776637, Y, Y
anti-Myc-tag , 1, Rb, MycTag, IHC-Fr, IP, WB, IHC-P, ICC, Electron Microscopy, WB, AB9106, n/a, 1:500, Abcam, 33534797, N, Y
anti-monomeric NeonGreen, 1, M, mNeonGreen fluorescent protein derived from Branchiostoma lanceolatum, IF, ELISA, WB, 32F6-100, 32F6, 1:1000, Chromotek, 32014414, Y, Y
anti-phospho-eIF2α, 1, Rb, H M R Mk Dm, WB, IP, IHC, WB, 3398, D9G8, 1:1000, Cell Signaling Technologies, 34953853, N, Y
anti-eIF2α, 1, Rb, H M R Mk, WB, IP, IHC, WB, 5324, D7D3, 1:1000, Cell Signaling Technologies, 34953853, N, Y

anti-Actin Antibody, 1, M, All, ELISA, ICC, IHC, IH(P), WB, WB, MAB1501R, C4, 1:10000, Millipore, 25753659, N, Y
anti-GFAP, 1, Rb, All, IHC, IHC, Z0334, n/a, 1:500, DAKO/Agilent, 11379820, N, Y
anti-F4/80, 1, R, M, F IHC IF IP WB , IHC, MCA497G, Cl:A3-1, 1 µg/mL, Serotec/BioRad, 31189648, N, Y
anti-human F(ab')2-alkaline phosphatase conjugated antibody, 2, G, H, IHC, ELISA, WB, ELISA, SAB3701239, n/a, 1:5000, Sigma, 32776637, N, Y
Streptavidin/HRP, 2, Streptomyces avidinii, Biotin, ELISA, ELISA, 554066, n/a, 1:1000, BD Biosciences, 20506300, N, Y
anti-mouse IgG, 2, Goat, M, ELISA, ICC, IHC, WB, WB, 115-035-062, n/a, 1:10000, Jackson ImmunoResearch, 31640849, N, Y
anti-rabbit IgG, 2, Goat, R, ELISA, ICC, IHC, WB, WB, 111-035-045, n/a, 1:10000, Jackson ImmunoResearch, 30143626, N, Y
Alexa594-conjugated goat anti-rabbit IgG , 2, G, R, IHC, IHC, A-11012, n/a, 1:1000, Thermo Fisher, 35190564, N, Y
Alexa594-conjugated rabbit anti-goat IgG , 2, Rb, G, IHC, IHC, A-11037, n/a, 1:1000, Thermo Fisher, 35600918, N, Y
Alexa647-conjugated goat anti-rat IgG , 2, G, R, IHC, IHC, A-21247, n/a, 1:1000, Thermo Fisher, 34211179, N, Y
Alexa488-conjugated goat anti-mouse IgG, 2, G, M, IHC, IHC, A-28175, n/a, 1:250, 1.6 µg/mL, Thermo Fisher, 34855620, N, Y
anti-NeuN Antibody,Alexa Fluor®488 conjugated, 1+2, M, H M R, IHC, IHC, MAB377X, A60, 1:1000, Merck, 32776637, N, Y

Species abbreviations:
H-Human
M-Mouse
R-Rat
Mk-Monkey
Dm-D. melanogaster
Rb-Rabbit
All-All Species Expected

Applications abbreviations:
ELISA-Enzyme-linked immunosorbent assay
WB-Western Blot
IP-Immunoprecipitation
IHC-Immunohistochemistry
IF-Immunofluorescence
F-Flow Cytometr
ICC-Immunocytochemistry
IEP-Immunoelectrophoresis
RID-Radial Immunodiffusion

## Eukaryotic cell lines

Policy information about cell lines and Sex and Gender in Research

Cell line source(s)

CAD5 cells were derived from Cath.a-differentiated (CAD) cells (Qi Y et al., J Neurosci 98), were established by Mahal et al., PNAS 2007 and were a kind gift from Charles Weissmann. Generation of CAD5 Prnp knock-out cells was described in Bardelli et al., PLOS Pathogens 2018.

Authentication

CAD5 cells were not authenticated after reception from Charles Weissmann.

Mycoplasma contamination

Cell lines were tested negative for mycoplasma contamination.

Commonly misidentified lines
(See ICLAC register)

No commonly misidentified cell lines were used in the study

## Animals and other research organisms

Policy information about studies involving animals; ARRIVE guidelines recommended for reporting animal research, and Sex and Gender in Research

Laboratory animals

Please specify the age for all strains of mice used, here in the reporting summary.

Please provide information on housing conditions for the mice, describing dark/light cycle, ambient temperature and humidity in the manuscript.
We used the following animals (both male and female) for slice culture and in vivo toxicity assessment: C57BL/6J, Tga20 (described in Fischer et al., EMBO J 1996). Additionally, Prnp0/0 (ZH1, described in Büeler et al., Cell 1993) and Prnp0/0 mice (ZH3, described in Nuvolone et al., J Exp Med 2016) were used for slice culture experiments. Mice were bred in high hygienic grade facilities and housed in groups of 3–5, under a 12 h light/12 h dark cycle (from 7 am to 7 pm) at 21±1°C, with sterilized food (Kliba No. 3431, Provimi Kliba, Kaiseraugst, Switzerland) and water ad libitum.

Animals were not selected for gender. Animals ages were as follows:
Slice culture: 9-12 days
MEMRI: 4 months
AAV and prion injection: 3 months

Wild animals

This study did not involve wild animals

Reporting on sex

We did not adjust for mouse gender because of lack of group-wise comparisons. Specifically, for Memri experiments, treatment and

| | |
|---|---|
| Reporting on sex | control were injected into the same animals. For AAV and prion inoculations, we compared survival versus antibody expression from only one group. |
| Field-collected samples | This study did not involve field-collected samples |
| Ethics oversight | We conducted all animal experiments in strict accordance with the Swiss Animal Protection law and dispositions of the Swiss Federal Office of Food Safety and Animal Welfare (BLV). The Animal Welfare Committee of the Canton of Zurich approved all animal protocols and experiments performed in this study (animal permits 123, ZH90/2013, ZH120/16, ZH139/16). |

Note that full information on the approval of the study protocol must also be provided in the manuscript.

# Flow Cytometry

## Plots

Confirm that:

☒ The axis labels state the marker and fluorochrome used (e.g. CD4-FITC).

☒ The axis scales are clearly visible. Include numbers along axes only for bottom left plot of group (a 'group' is an analysis of identical markers).

☒ All plots are contour plots with outliers or pseudocolor plots.

☒ A numerical value for number of cells or percentage (with statistics) is provided.

## Methodology

| | |
|---|---|
| Sample preparation | CAD5 cells were cultured with 20mL Corning Basal Cell Culture Liquid Media-DMEM and Ham's F-12, 50/50 Mix supplemented with 10% FBS, Gibco MEM Non-Essential Amino Ac-ids Solution 1X, Gibco GlutaMAX Supplement 1X and 0.5mg/mL of Geneticin in T75 Flasks ThermoFisher at 37°C 5% CO2. 16 hours before treatment, cells were split into 96wells plates at 25000 cells/well in 100µL.<br>POM1 alone was prepared at 5 µM final concentration, in 20 mM HEPES pH 7.2 and 150 mM NaCl. 100 µL of each sample, including buffer control, were added to CAD5 cells, in duplicates.<br>After 48 hours, cells were washed two times with 100µL MACS buffer (PBS + 1% FBS + 2 mM EDTA) and resuspended in 100 µL MACS buffer. 30'' before FACS measurements PI (1 µg/mL) was added to cells |
| Instrument | BD LSRFortessa |
| Software | FlowJo (10) |
| Cell population abundance | Only CAD5 cells are present in the sample |
| Gating strategy | Based on FSC and SSC the CAD5 cells were selected and separated from debris. After the first gating the cells were analyzed for PI presence. The gate to discriminate PI positive and negative cells was selected on control samples (not treated) and applied to all the other samples. A figure exemplifying the gating strategy is provided in ED Figure 3A. |

☒ Tick this box to confirm that a figure exemplifying the gating strategy is provided in the Supplementary Information.

# Magnetic resonance imaging

## Experimental design

| | |
|---|---|
| Design type | No BOLD imaging / fMRI |
| Design specifications | No BOLD imaging / fMRI |
| Behavioral performance measures | No behavioural performance measures were aquired. |

## Acquisition

| | |
|---|---|
| Imaging type(s) | Diffusion weighted imaging |
| Field strength | 4.7 |
| Sequence & imaging parameters | TR: 300 ms TE: 28 ms, flip angle: 90 deg, average: 1, Matrix: 350 x 350, Field of View: 3 x 3 cm, acquisi-tion time: 17 min, voxel size: 87x87 µm3, slice thickness: 700 µm3, Isodistance: 1400 µm3 and b values: 13, 816 s/mm2 |
| Area of acquisition | Converging on the whole hippocampus |

Diffusion MRI ☒ Used ☐ Not used

| | |
|---|---|
| Parameters | Single shell, b values: 13, 816 s/mm2 |

## Preprocessing

| | |
|---|---|
| Preprocessing software | Biomap software |
| Normalization | *If data were normalized/standardized, describe the approach(es): specify linear or non-linear and define image types used for transformation OR indicate that data were not normalized and explain rationale for lack of normalization.* |
| Normalization template | n/a |
| Noise and artifact removal | n/a |
| Volume censoring | n/a |

## Statistical modeling & inference

| | |
|---|---|
| Model type and settings | no fMRI |
| Effect(s) tested | no fMRI |

Specify type of analysis: ☐ Whole brain ☐ ROI-based ☐ Both

| | |
|---|---|
| Statistic type for inference (See Eklund et al. 2016) | no fMRI |
| Correction | no fMRI |

## Models & analysis

| n/a | Involved in the study |
|---|---|
| ☒ | ☐ Functional and/or effective connectivity |
| ☒ | ☐ Graph analysis |
| ☒ | ☐ Multivariate modeling or predictive analysis |
