# Supplementary figures and images for: A conformational switch controlling the toxicity of the prion protein
Source: Nat Struct Mol Biol. 2022 Aug 10;29(8):831–40. doi: 10.1038/s41594-022-00814-7 (PMC9371974; doi:10.1038/s41594-022-00814-7)

# Fig2D

Page 1 – raw gel

Page 2 – annotation, ladder

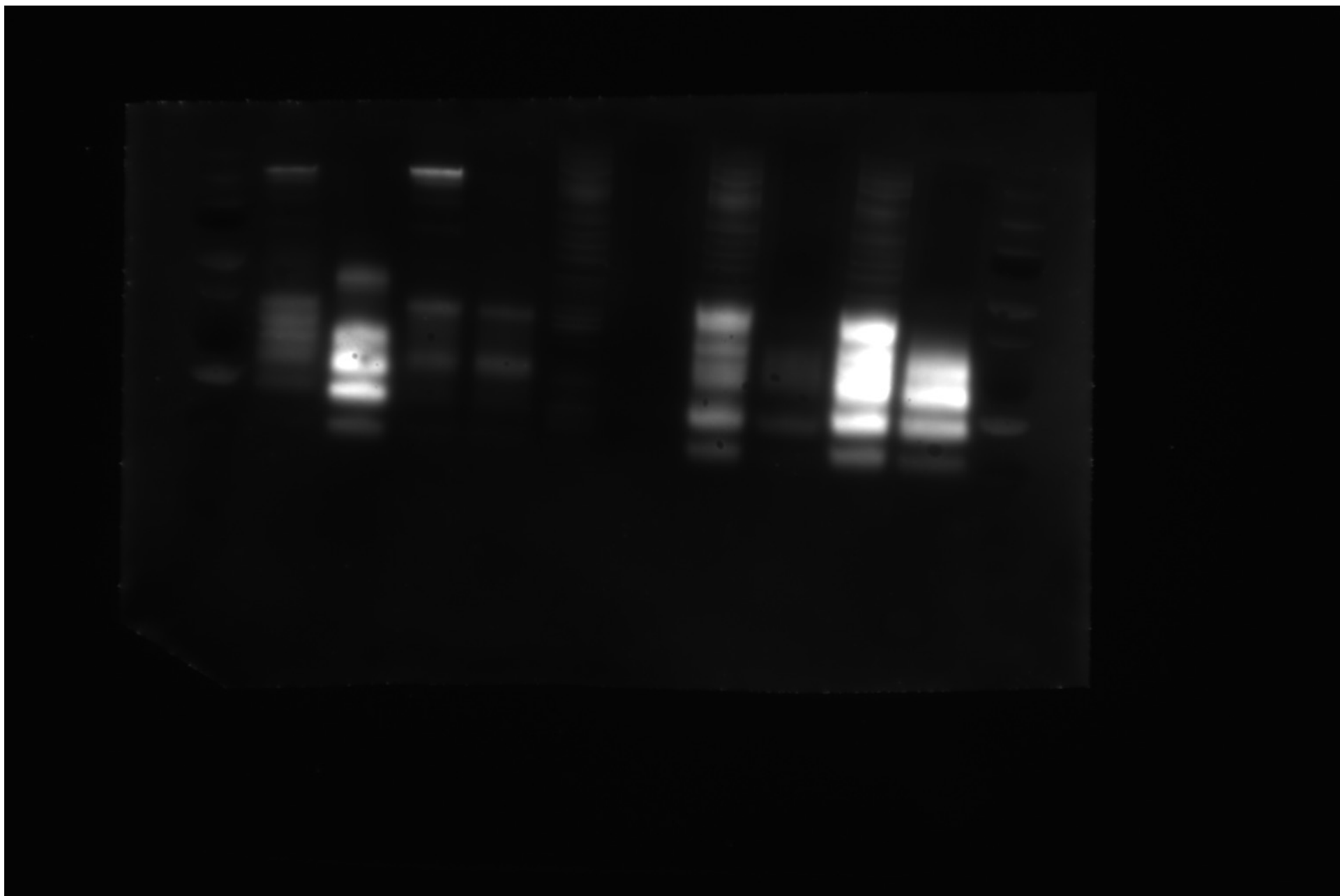

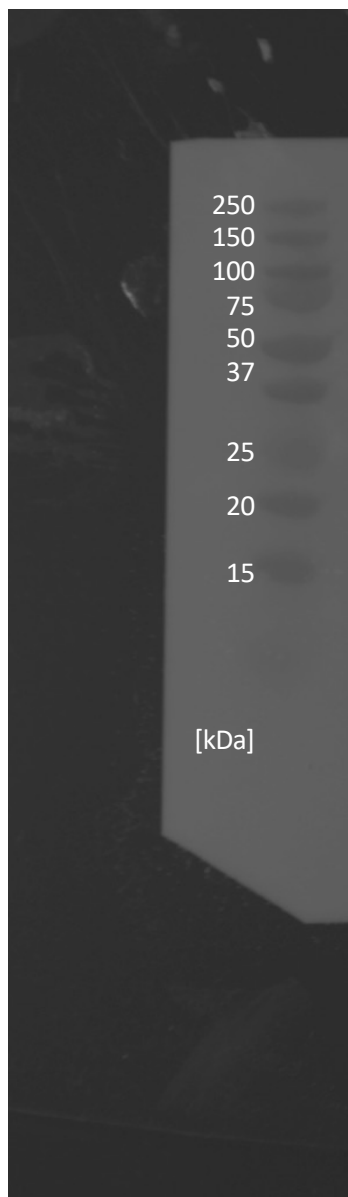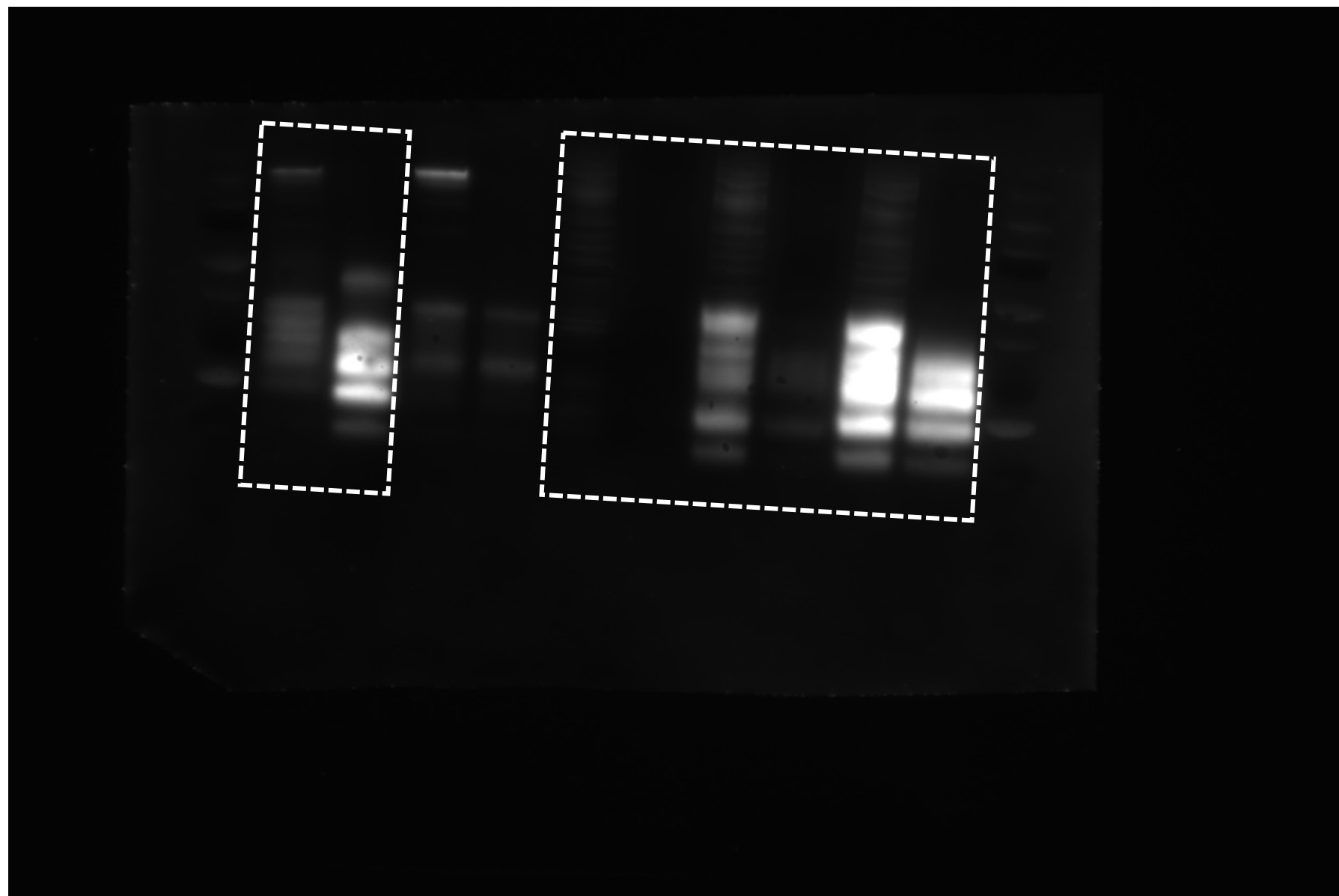

Supplement: Source Data Fig. 2 — Unprocessed Western Blots [file 41594_2022_814_MOESM7_ESM.pdf]

# Fig 4F

Page 1 – raw gel

Page 2 – annotation, ladder

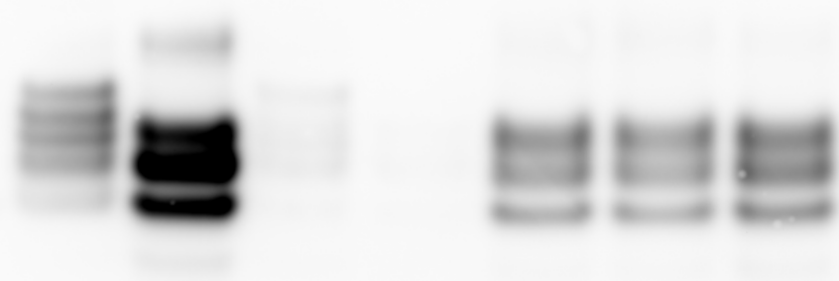

Fig 4F

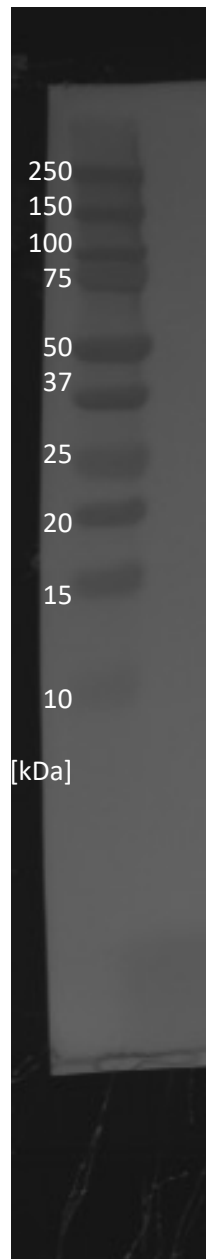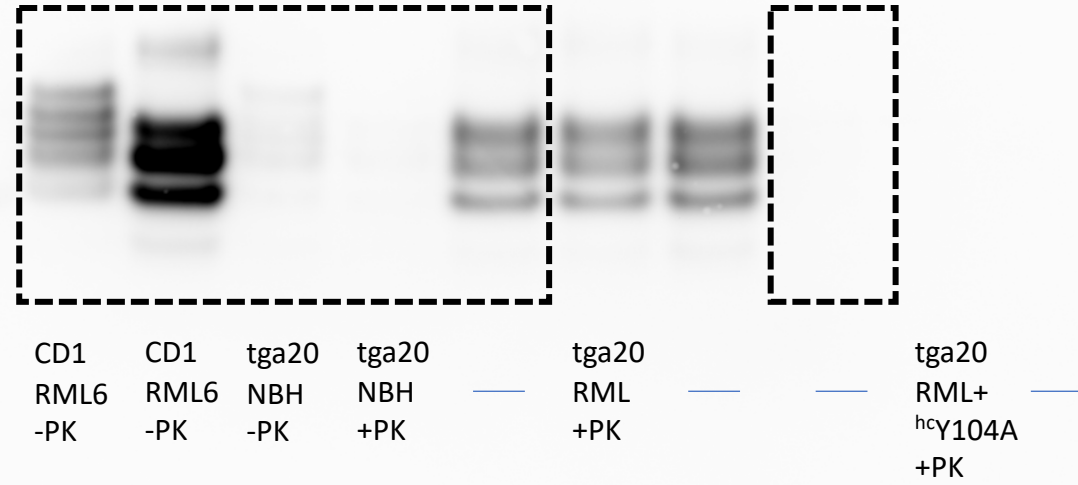

Supplement: Source Data Fig. 4 — Unprocessed Western Blots [file 41594_2022_814_MOESM9_ESM.pdf]
