# Supplementary material for: A conformational switch controlling the toxicity of the prion protein
Source: Nat Struct Mol Biol. 2022 Aug 10;29(8):831–40. doi: 10.1038/s41594-022-00814-7 (PMC9371974; doi:10.1038/s41594-022-00814-7)

Fig 6H

Page 1 – raw gel, mycTag

Page 2 – raw gel, pan Actin

Page 3 – annotation, ladder, mycTag

Page 4 – annotation, ladder, pan Actin

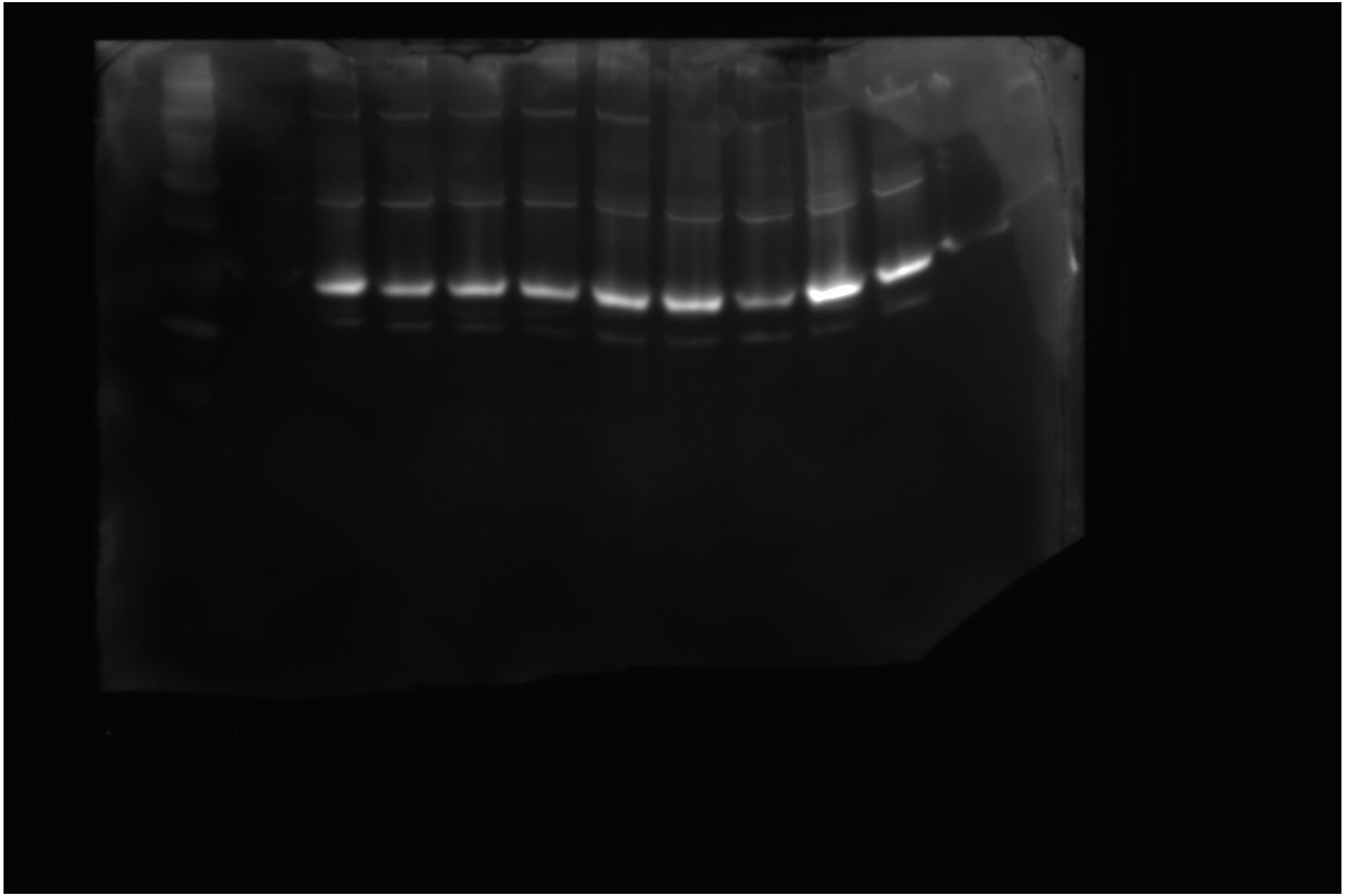

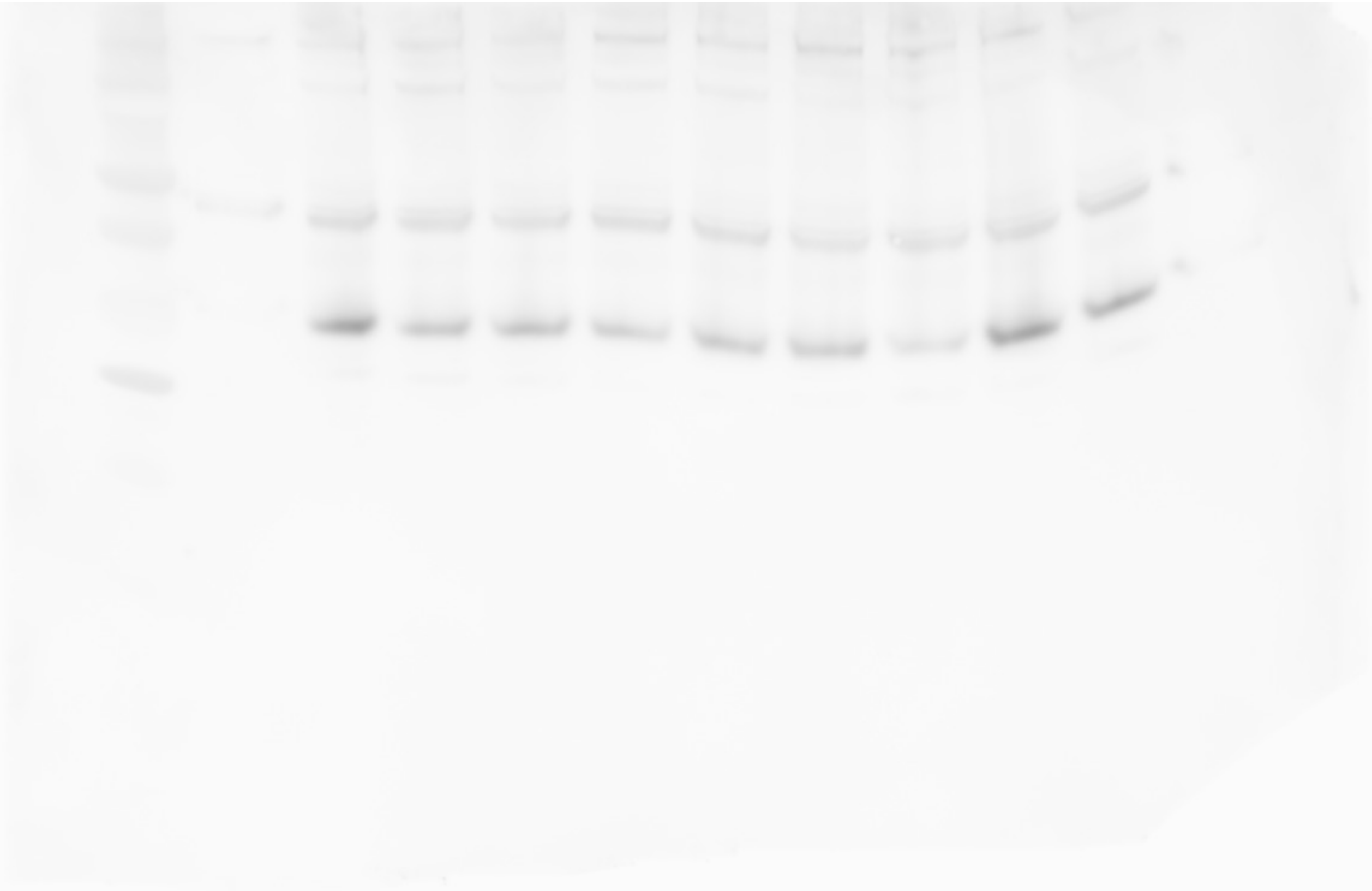

Fig 6H – myc-tag

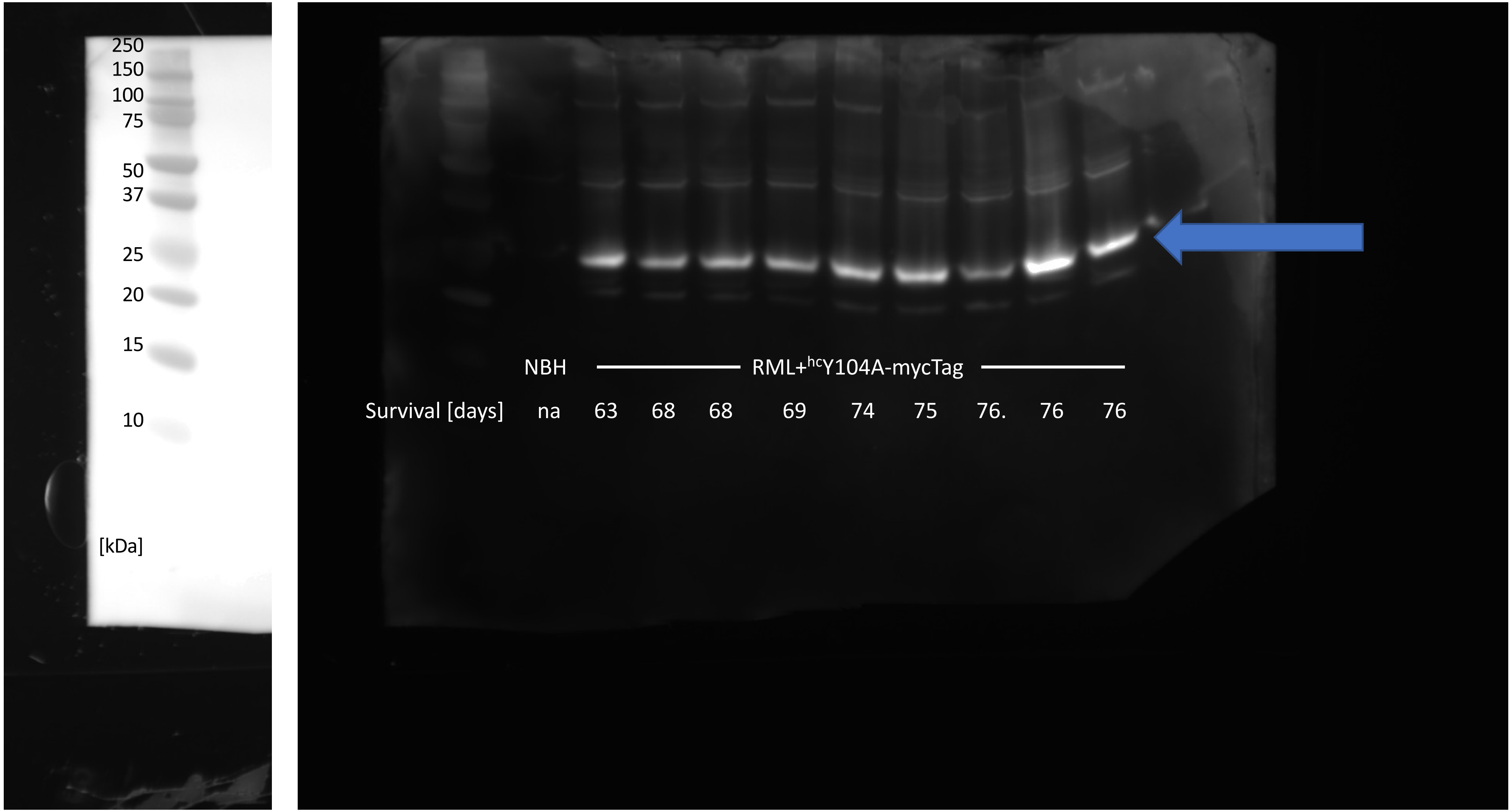

Fig 6H – Pan actin

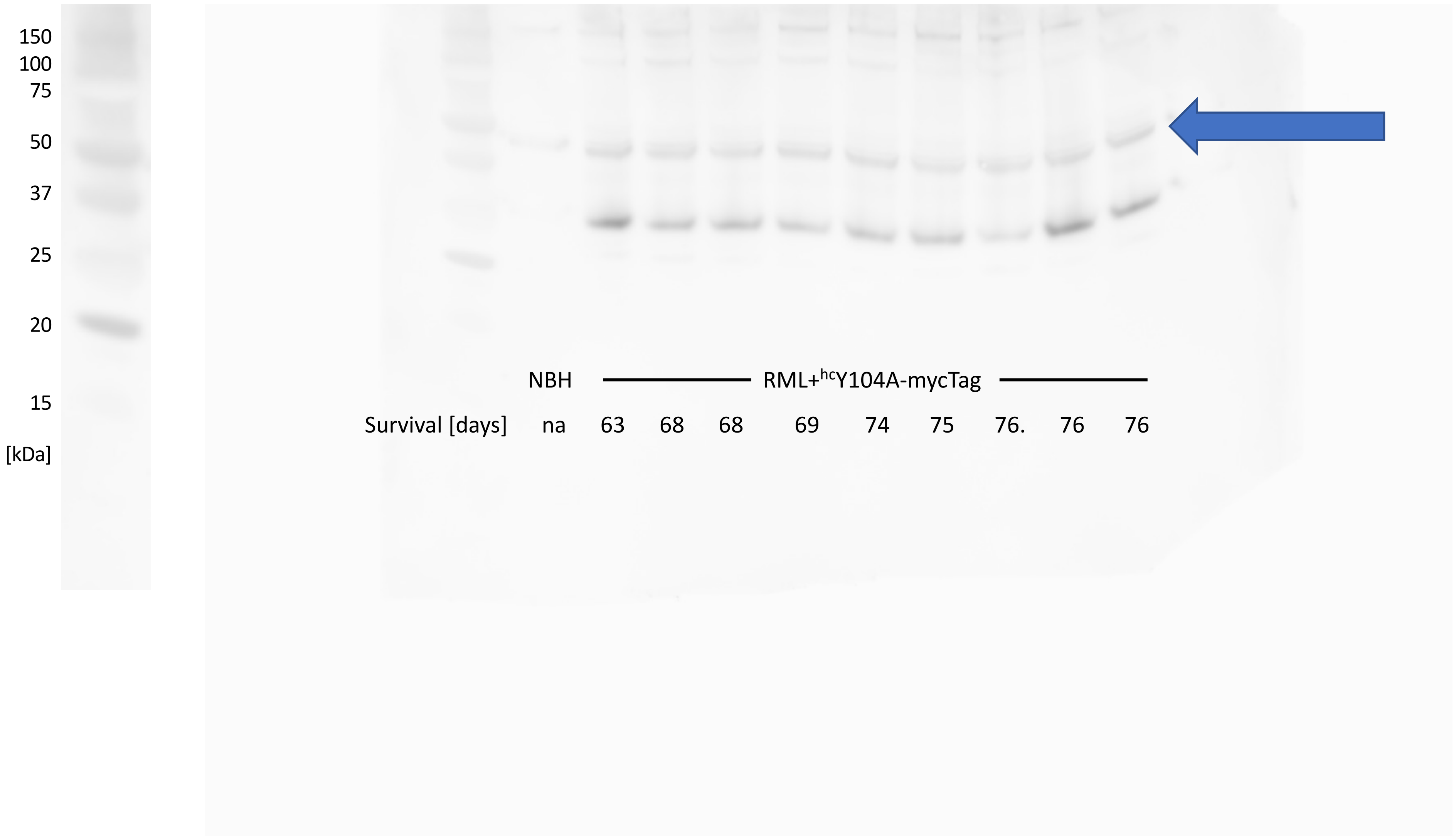

Supplement: Source Data Fig. 6 — Unprocessed Western Blots [file 41594_2022_814_MOESM11_ESM.pdf]
