# Supplementary material for: A conformational switch controlling the toxicity of the prion protein
Source: Nat Struct Mol Biol. 2022 Aug 10;29(8):831–40. doi: 10.1038/s41594-022-00814-7 (PMC9371974; doi:10.1038/s41594-022-00814-7)
Supplement: Source Data Extended Data Fig. 2 — Unprocessed Western Blots [file 41594_2022_814_MOESM15_ESM.pdf]

Extended Data Fig. 2C

Page 1 – raw gel POM19

Page 2 – raw gel pan actin

Page 3 – annotation, ladder, POM19

Page 4 – annotation, ladder, pan actin

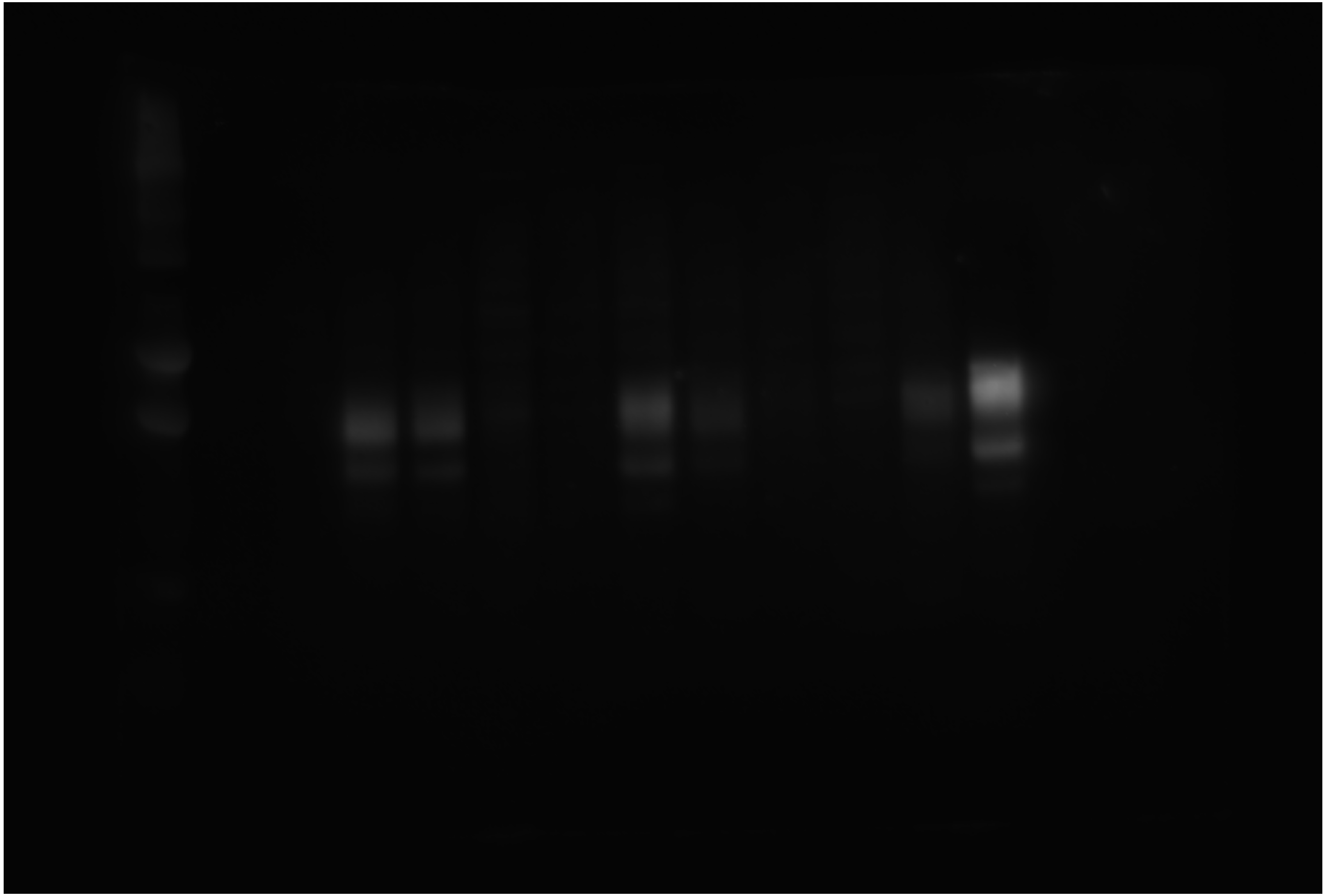

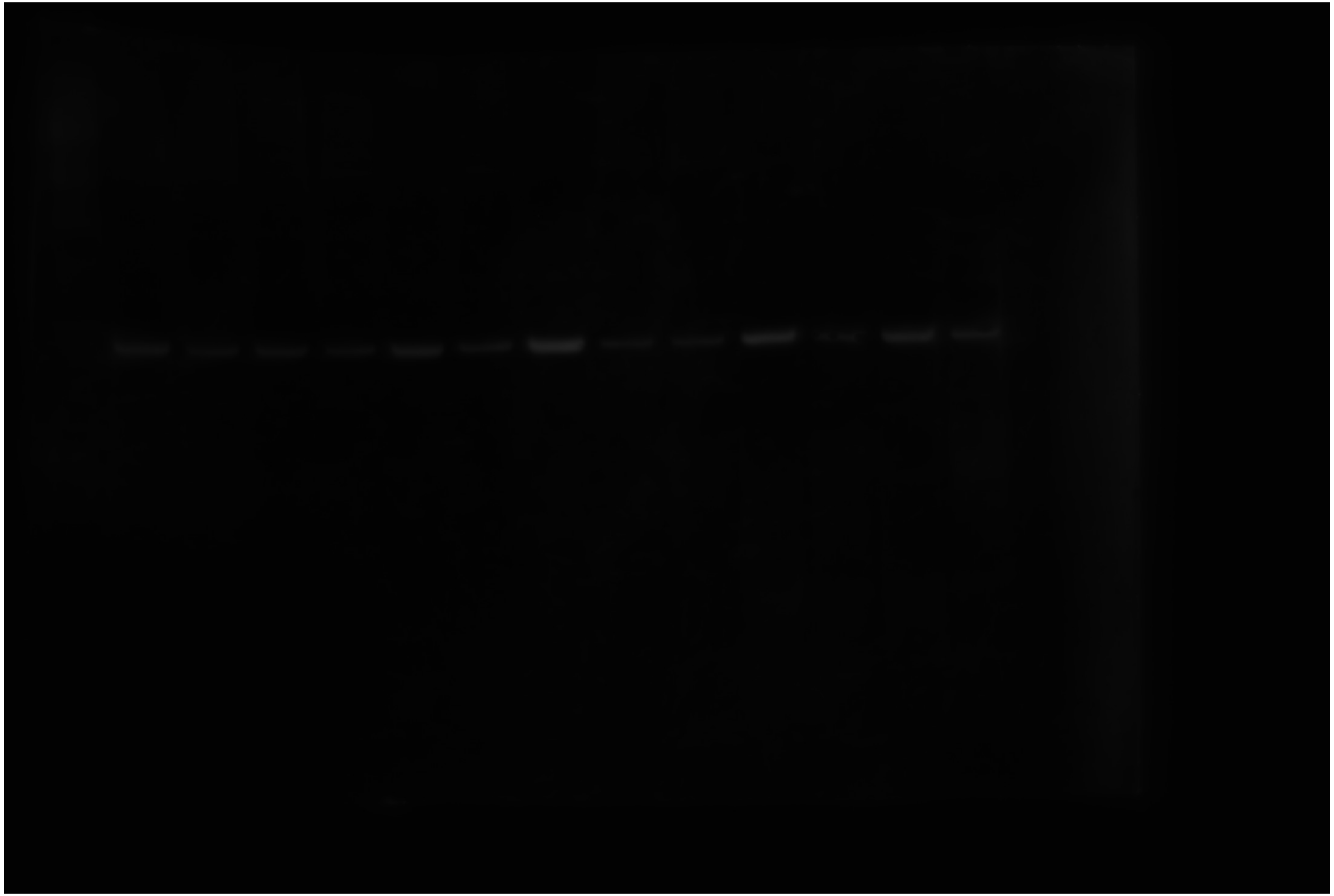

ED Fig 2C – POM19

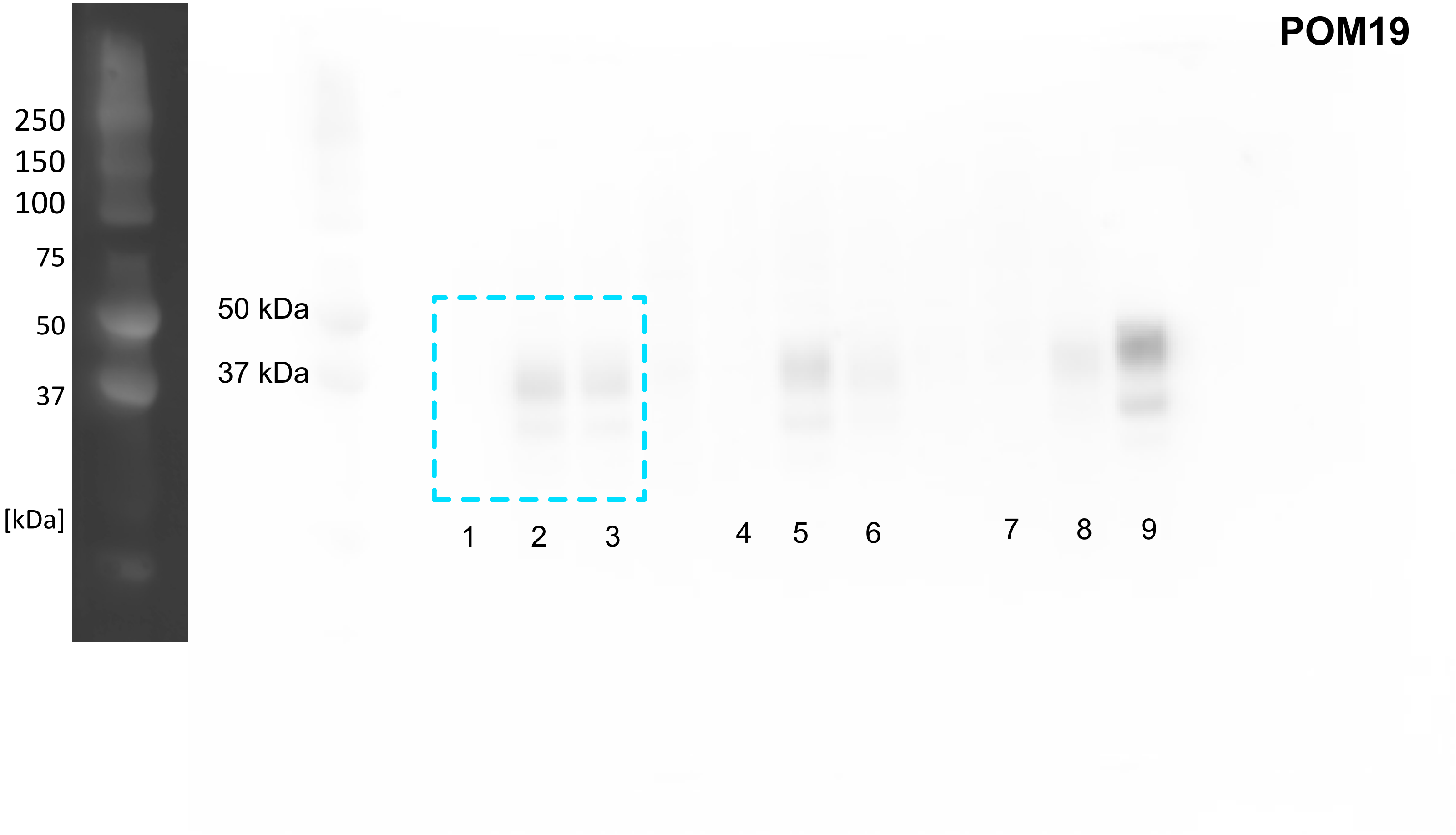

1, 4, 6: CAD5 + empty control plasmid
2, 5, 7: CAD5 + mPrP^{C}
3, 6, 9: CAD5 + mPrP^{R207A}

ED Fig 2C – actin

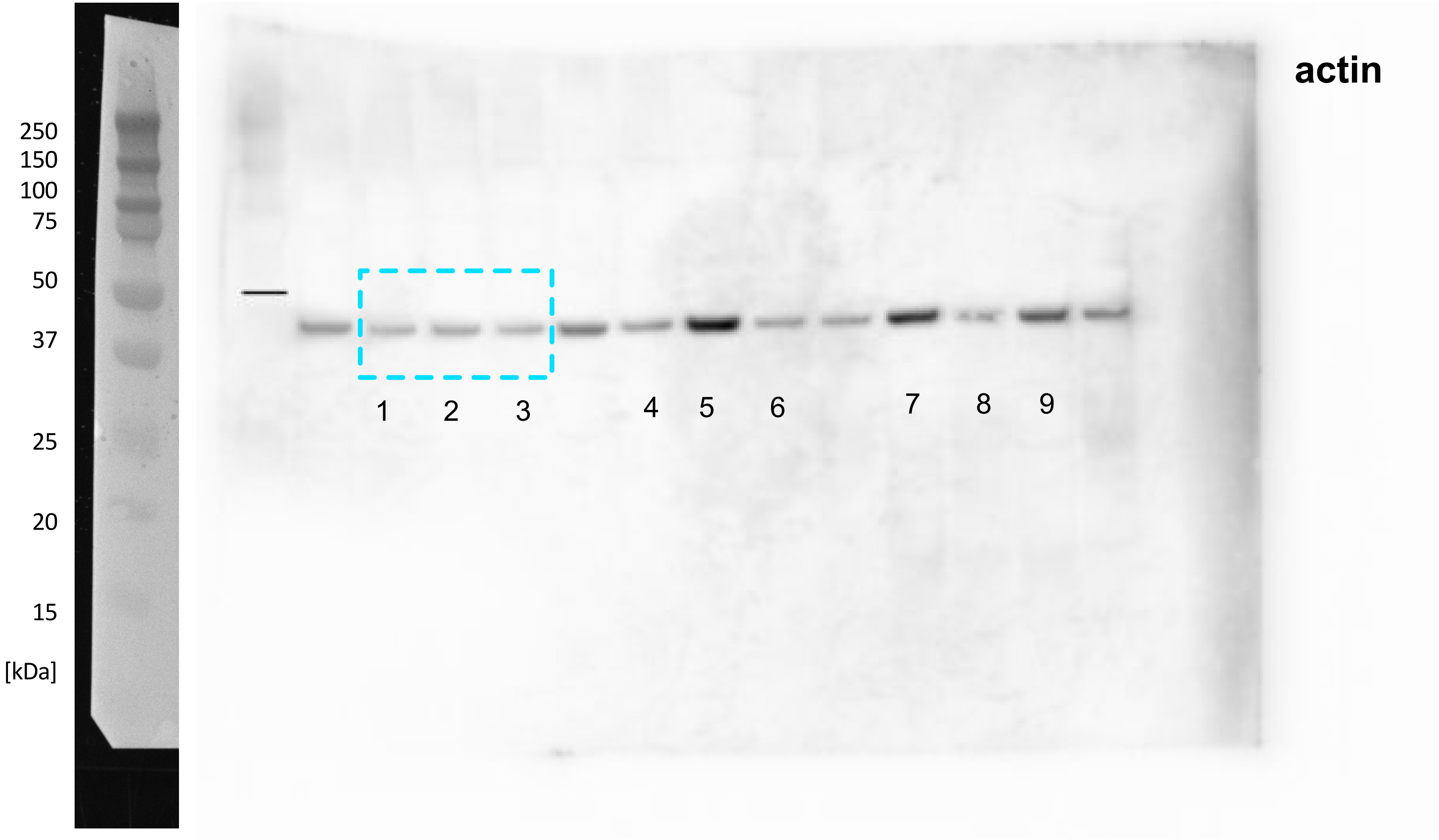

1, 4, 6: CAD5 + empty control plasmid
2, 5, 7: CAD5 + mPrP^{C}
3, 6, 9: CAD5 + mPrP^{R207A}
