# Supplementary material for: A conformational switch controlling the toxicity of the prion protein
Source: Nat Struct Mol Biol. 2022 Aug 10;29(8):831–40. doi: 10.1038/s41594-022-00814-7 (PMC9371974; doi:10.1038/s41594-022-00814-7)
Supplement: Source Data Extended Data Fig. 3 — Unprocessed Western Blots [file 41594_2022_814_MOESM17_ESM.pdf]

Extended Data Figure 3

Page 14	Figure	Description
1	ED Fig. 3B	POM2, raw gel
2	ED Fig. 3B	Pan actin, raw gel
3	ED Fig. 3B	POM2, annotated, ladder
4	ED Fig. 3B	Pan actin, annotated, ladder
5	ED Fig. 3C	peIF2a, raw gel
6	ED Fig. 3C	eIF2a, raw gel
7	ED Fig. 3C	Pan actin, raw gel
8	ED Fig. 3C	peIF2a, annotated, ladder
9	ED Fig. 3C	eIF2a, annotated, ladder
10	ED Fig. 3C	Pan actin, annotated, ladder
11	ED Fig. 3E	mNG, raw gel
12	ED Fig. 3E	Pan actin, raw gel
13	ED Fig. 3E	mNG, annotated, ladder
14	ED Fig. 3E	Pan actin, annotated, ladder

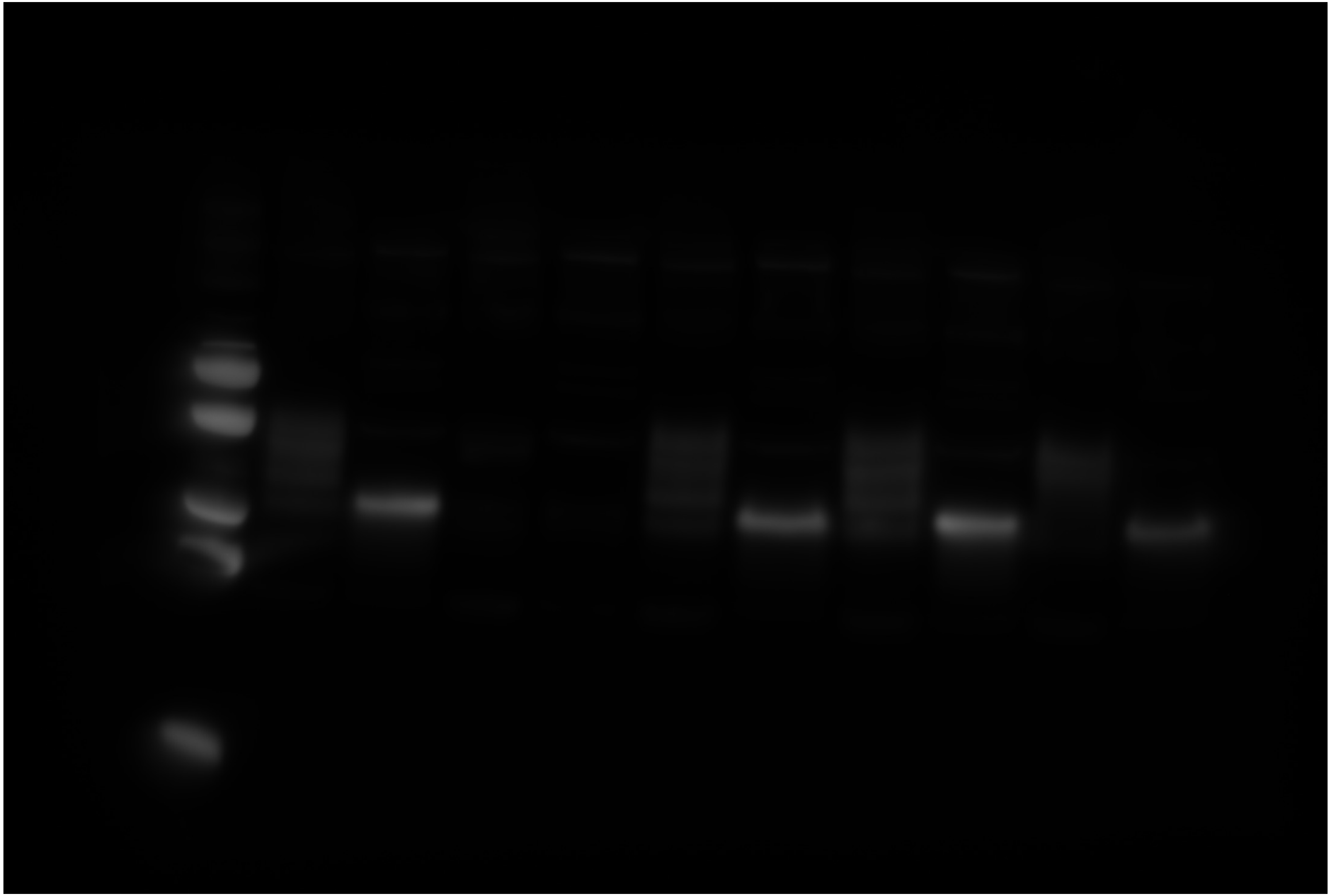

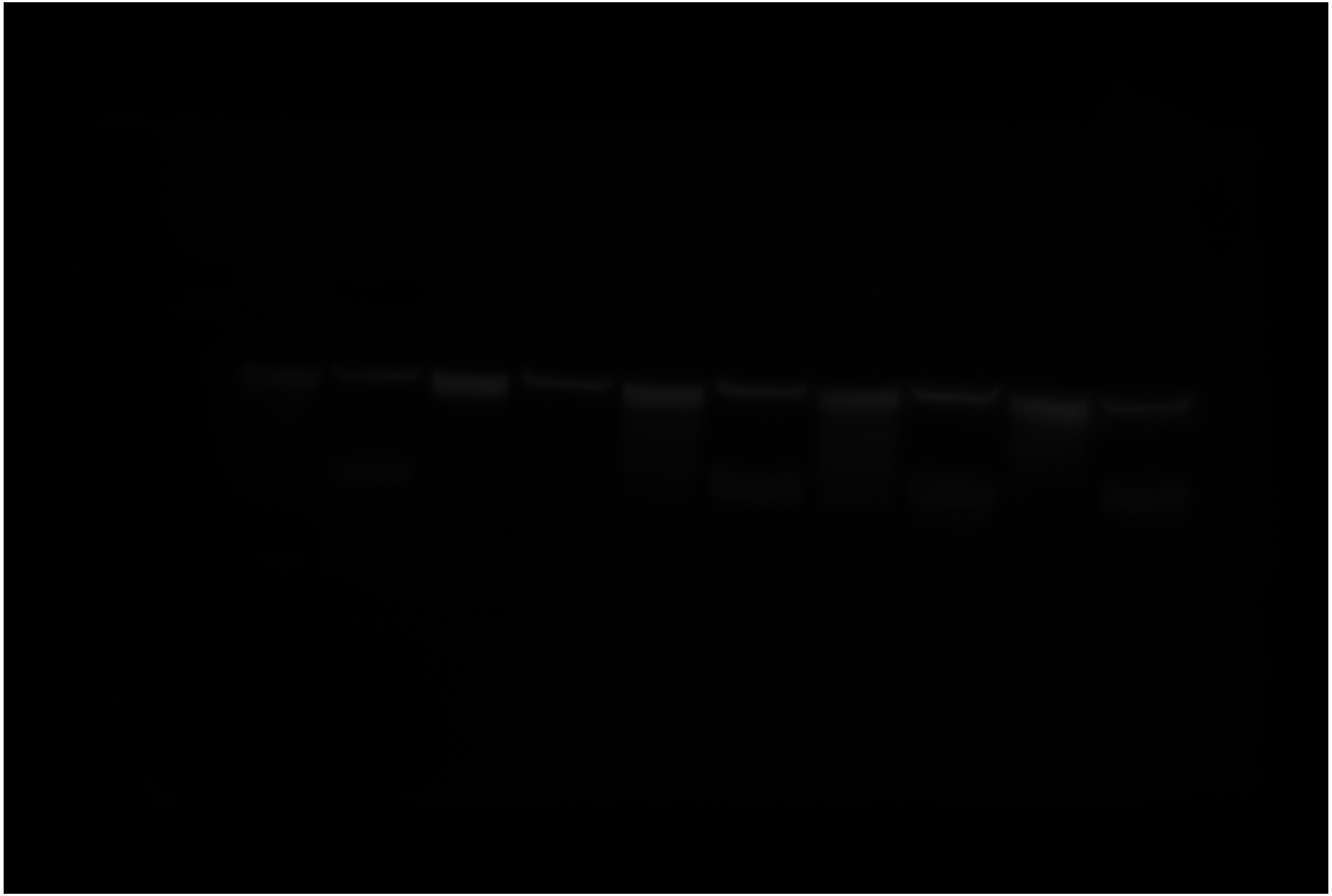

ED Fig 3B – POM2

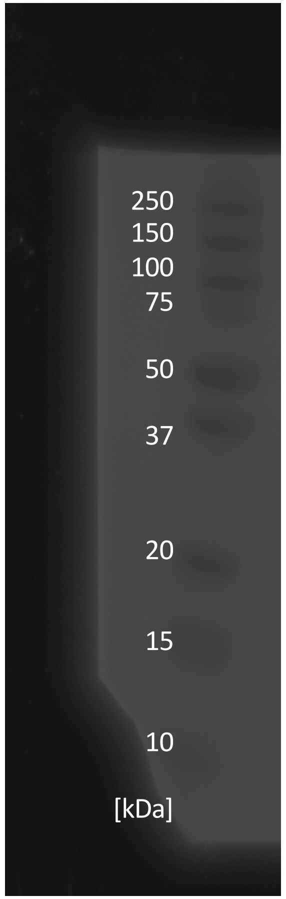

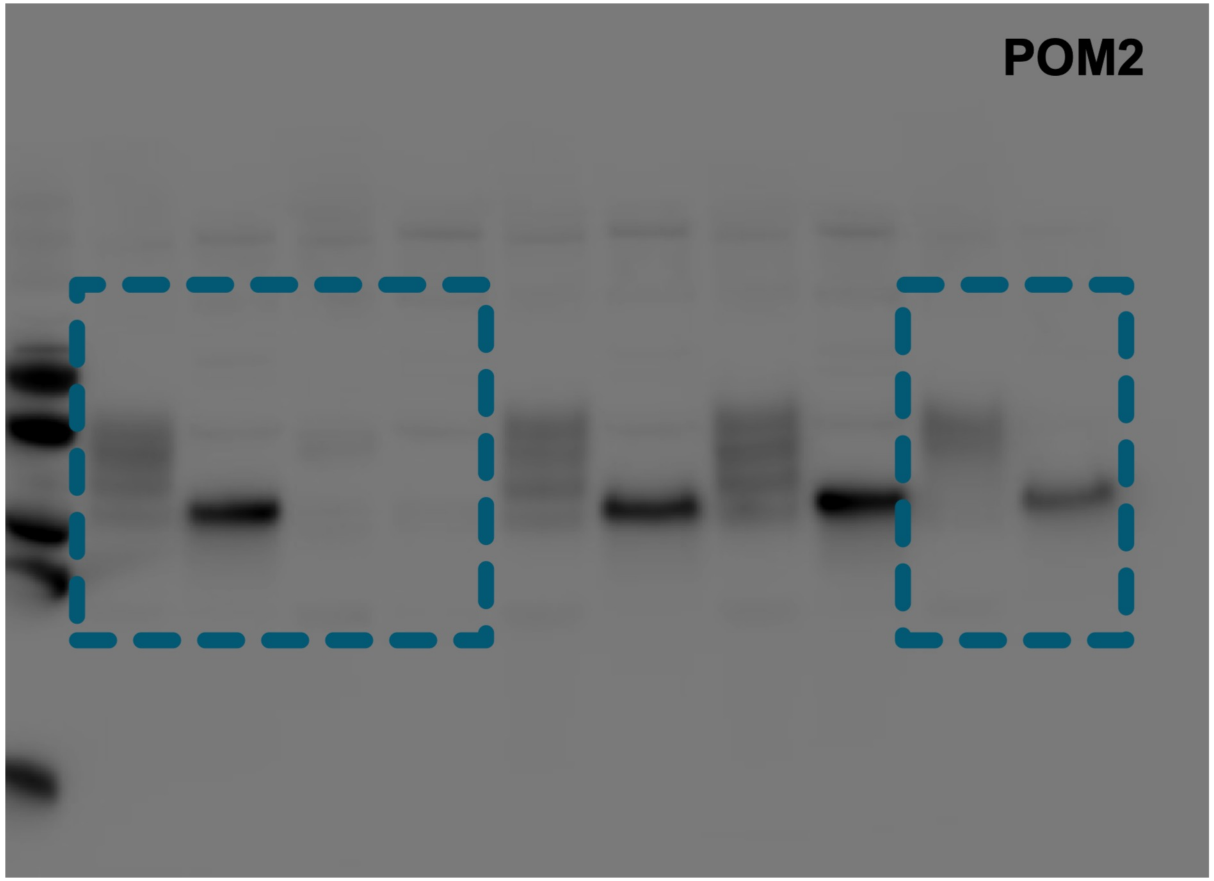

ED Fig 3B - actin

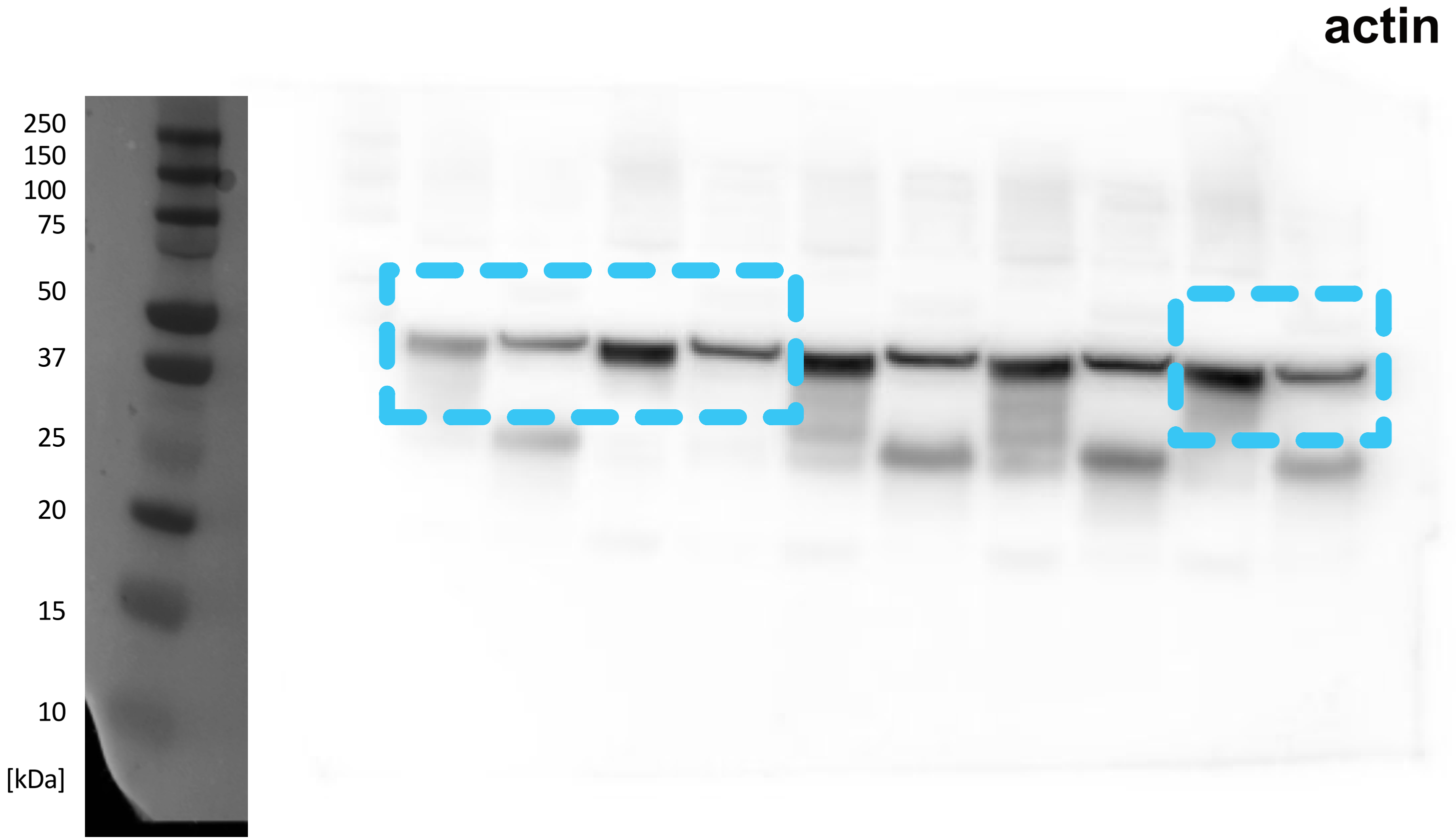

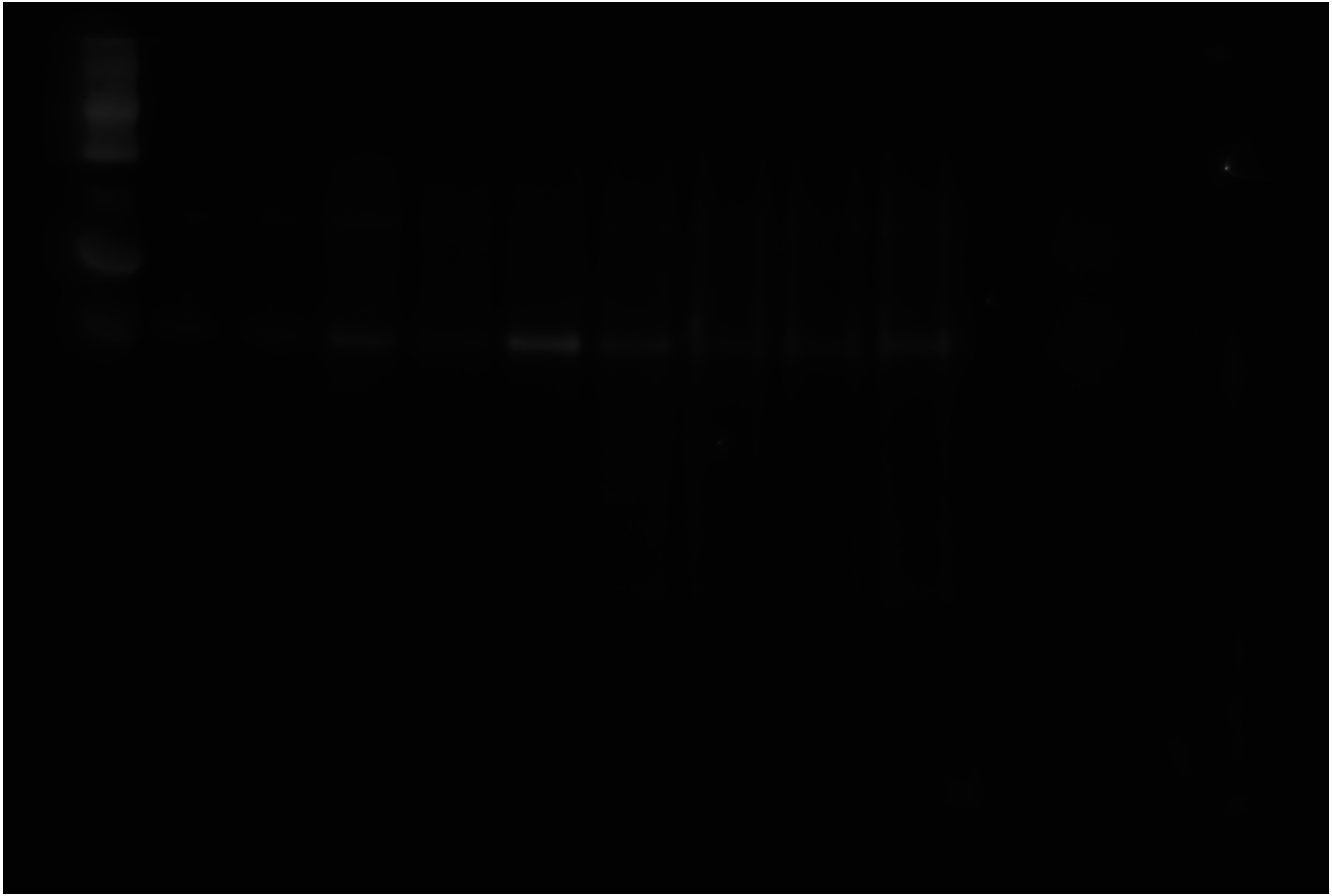

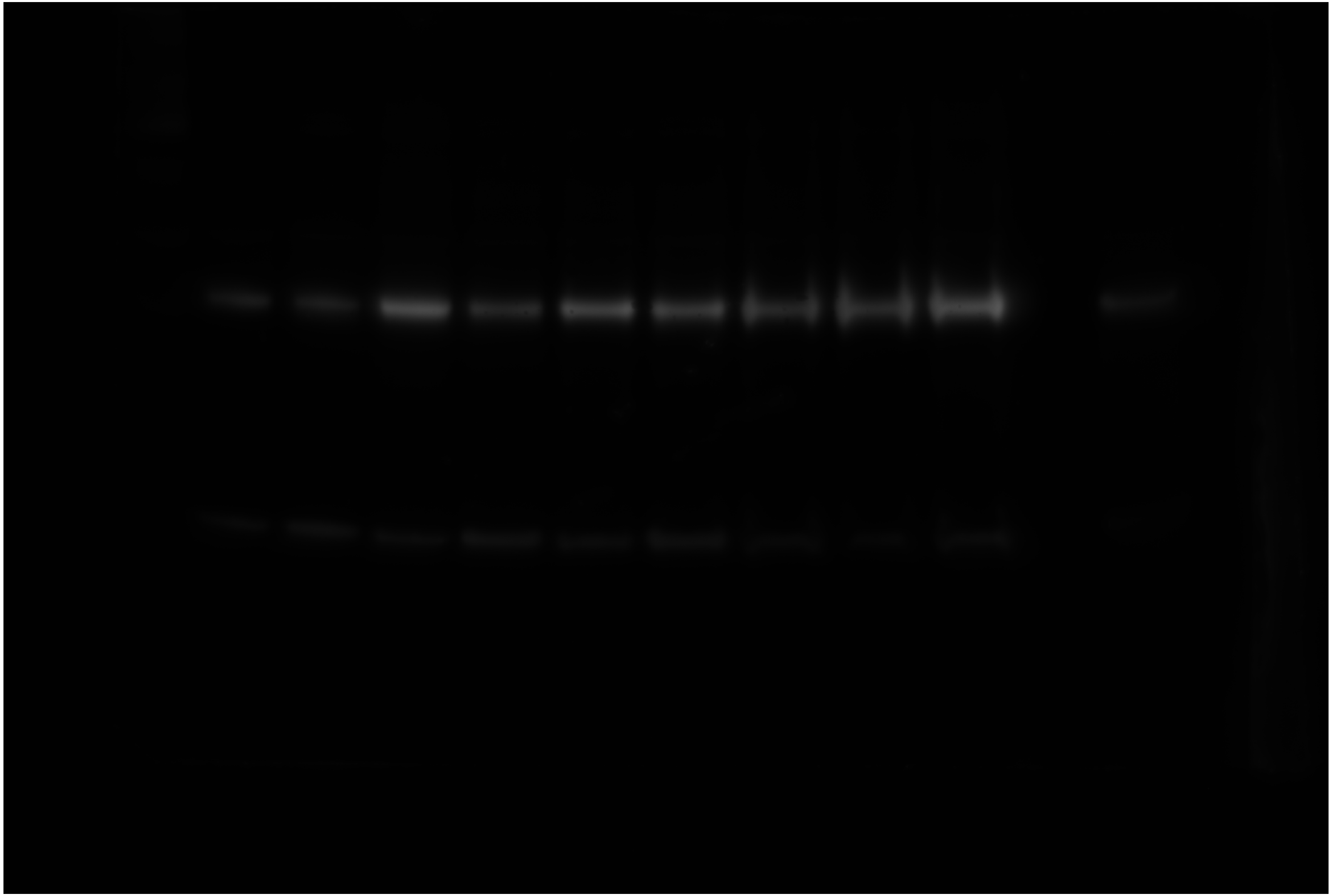

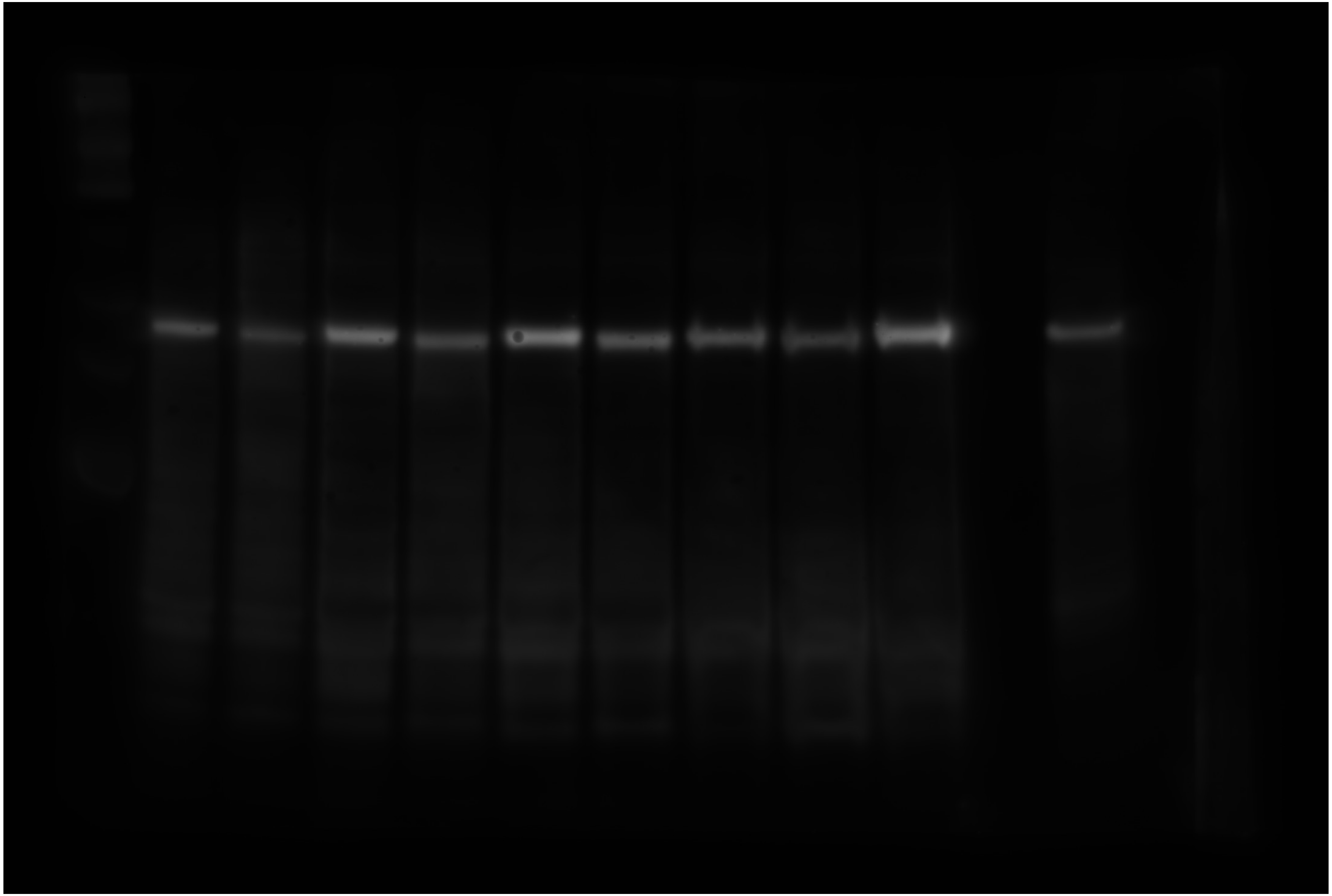

ED Fig 3C – peIF2a

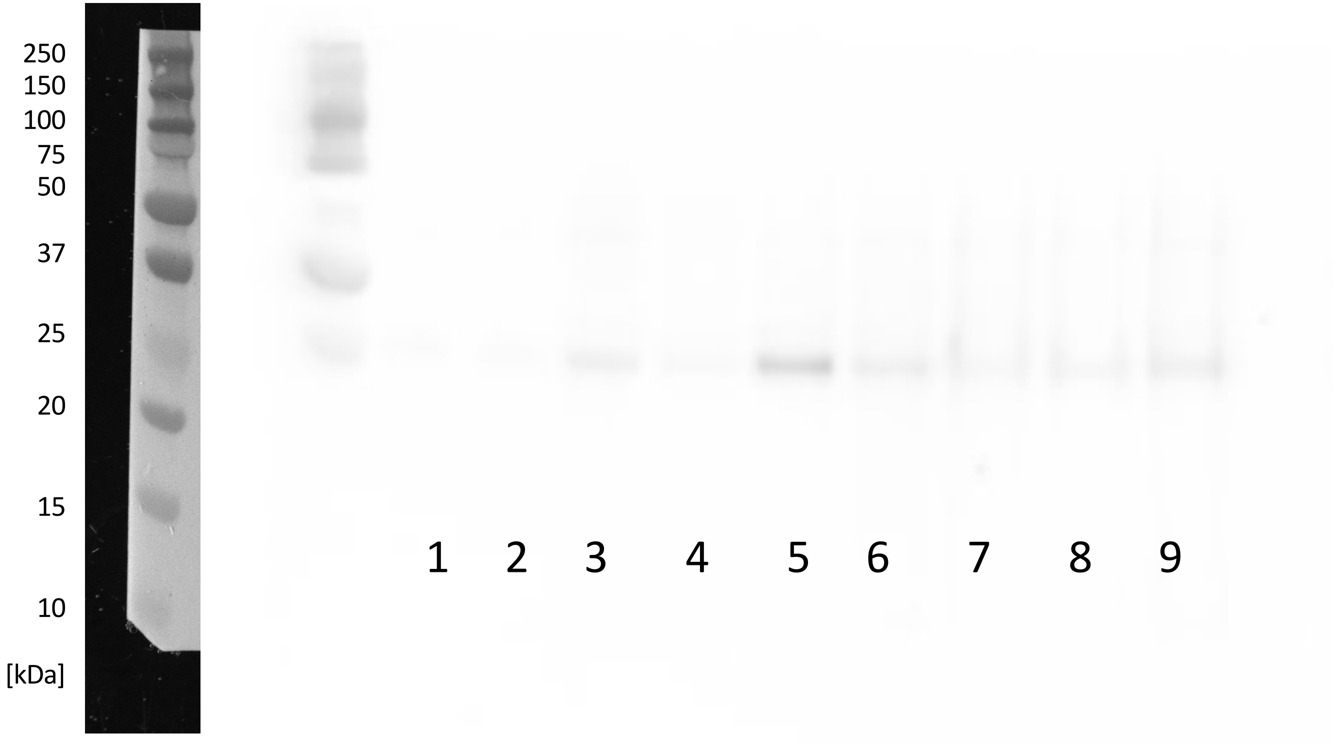

1-3 CAD5 + pcDNA3.1 empty ctrl
4-6 CAD5 + pcDNA3.1 wt ctrl
7-9 CAD5 + pcDNA3.1 I138C-R207C

ED Fig 3C – eIF2a

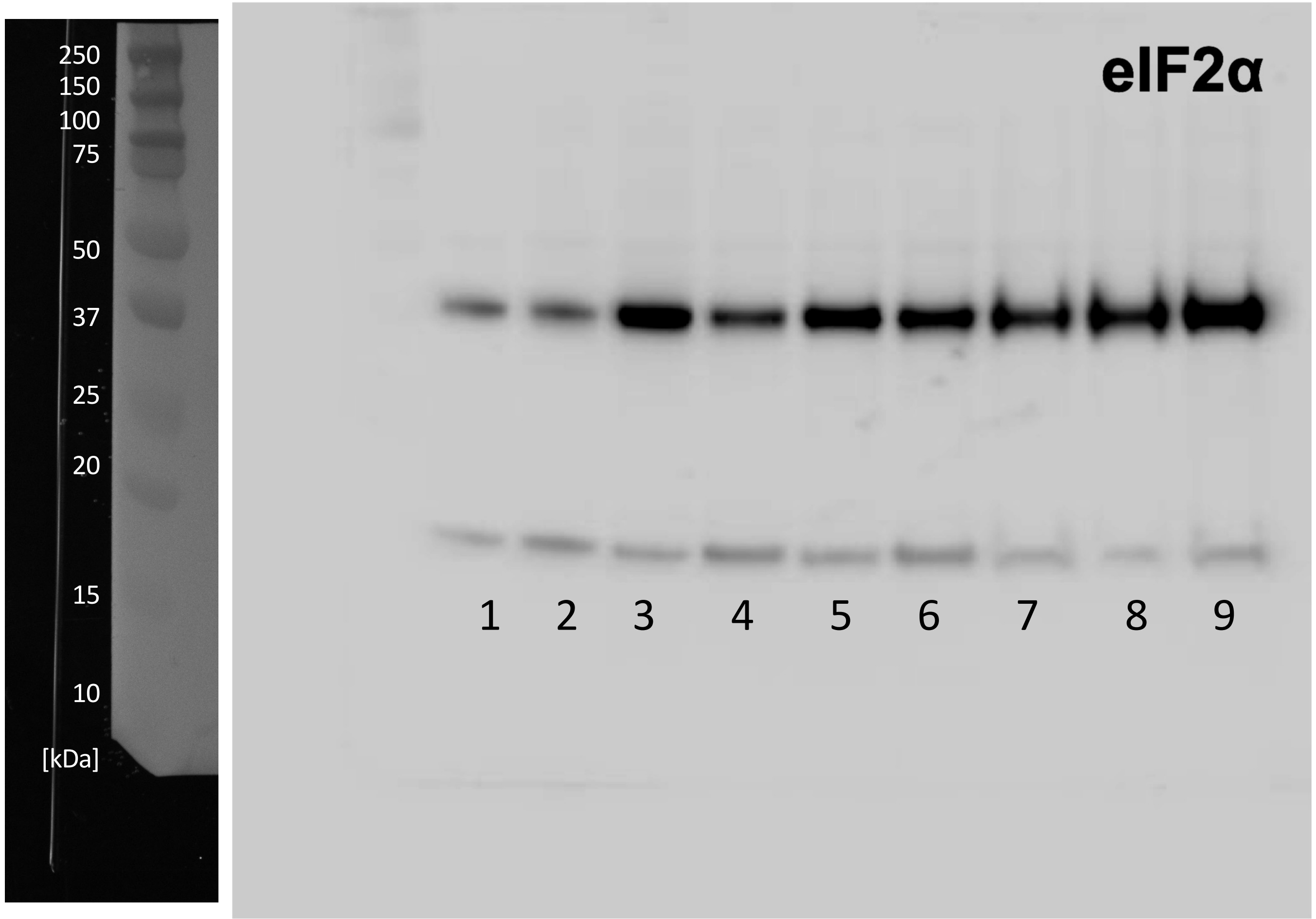

ED Fig 3C

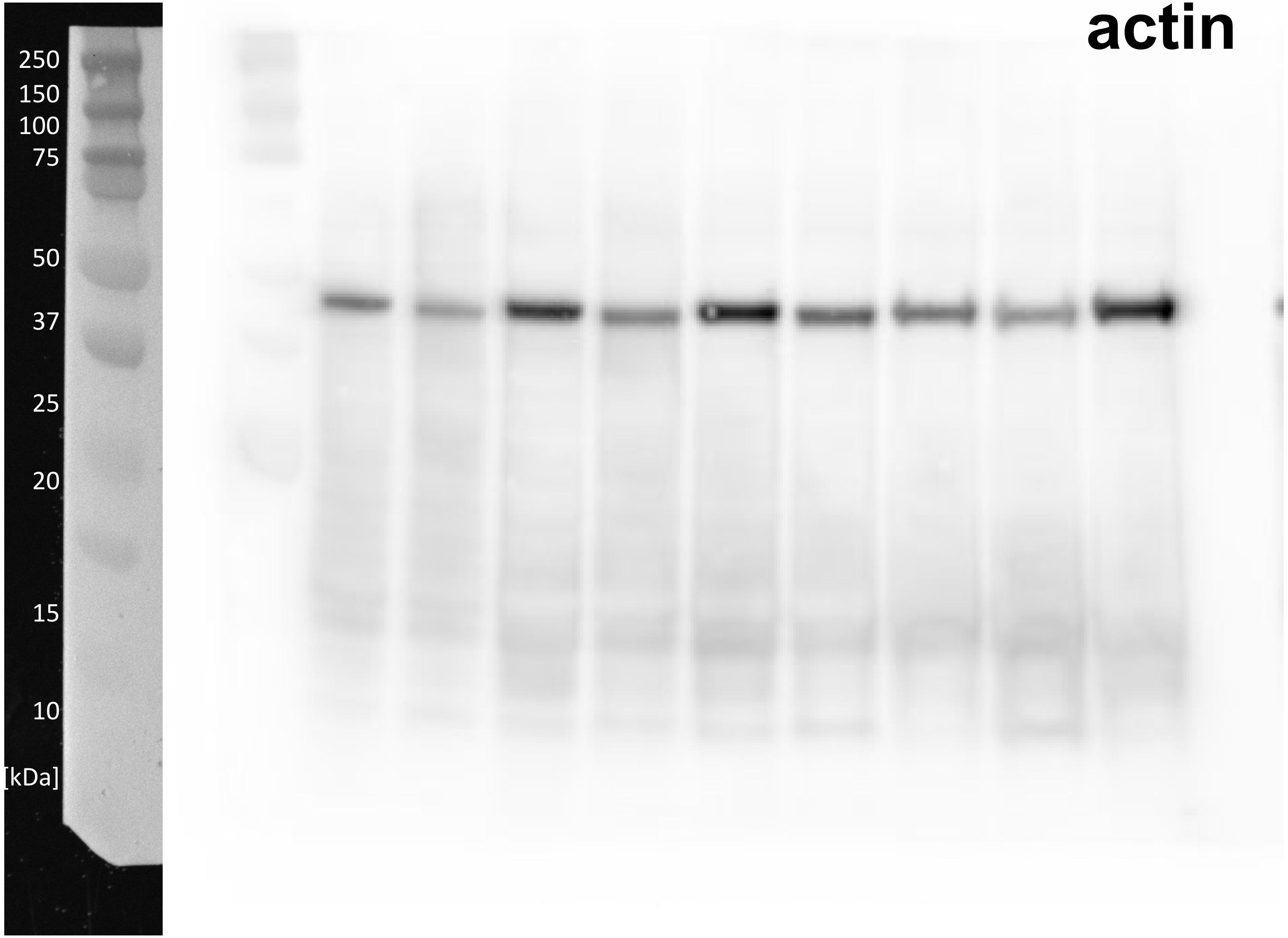

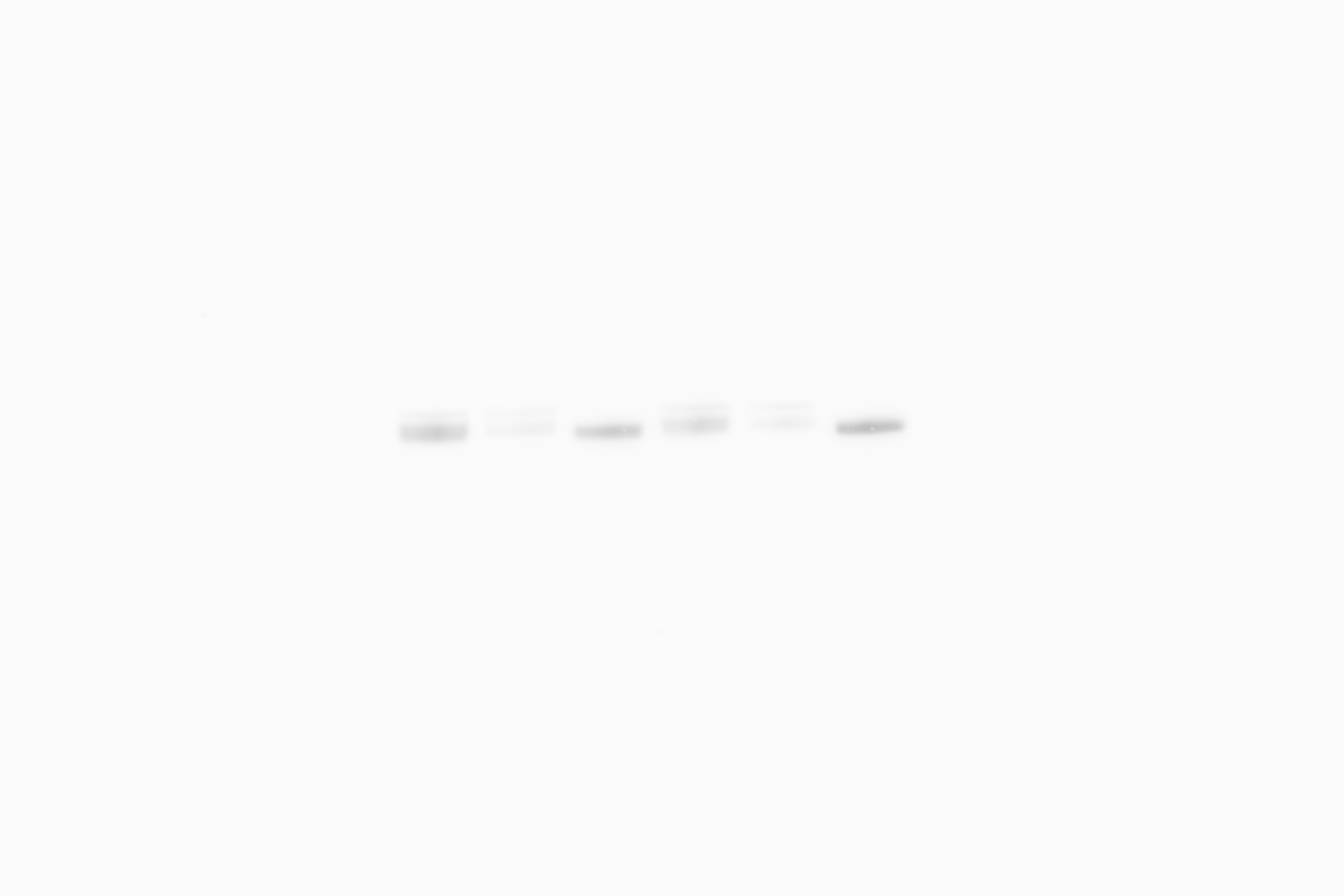

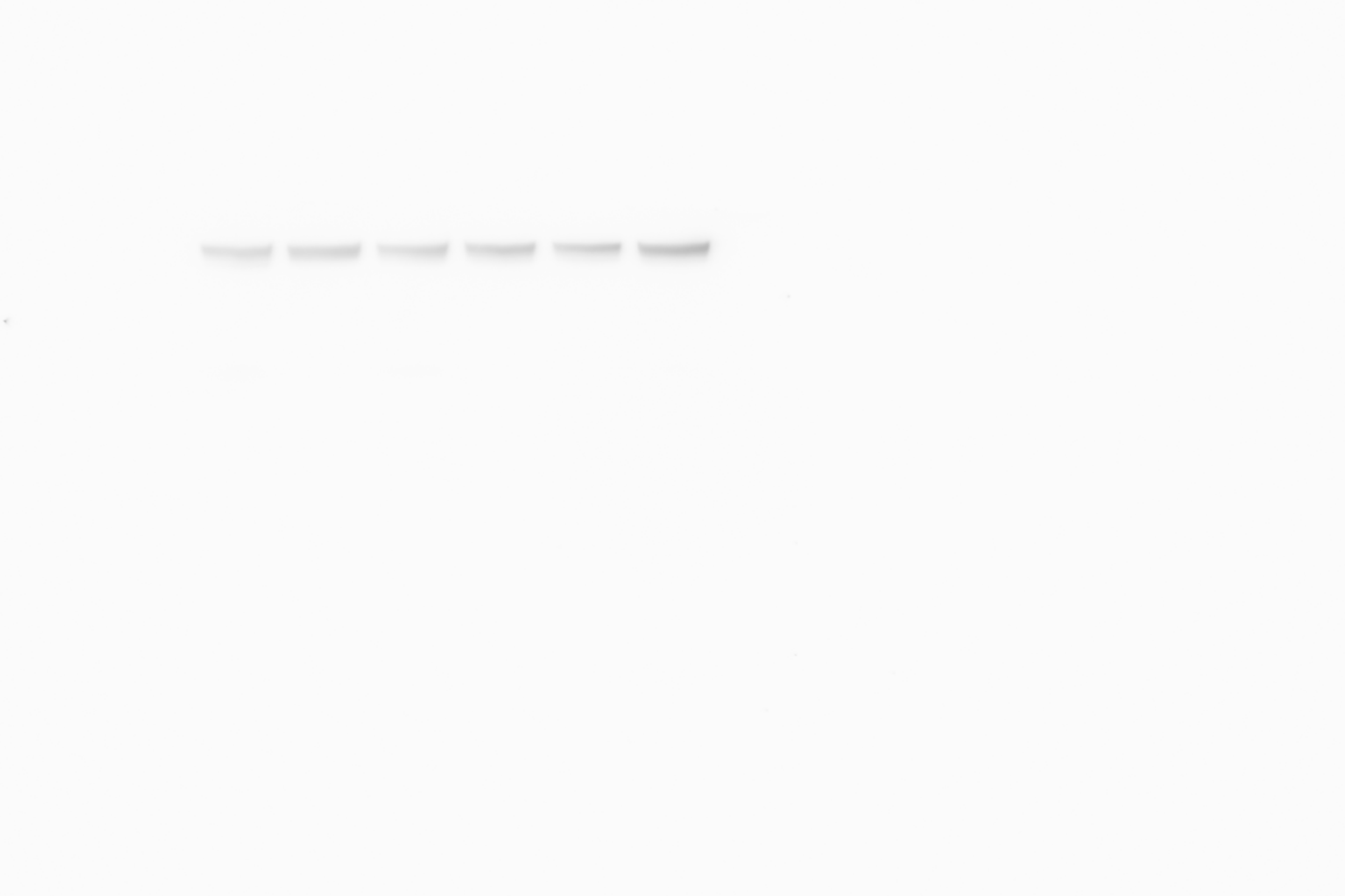

ED Fig 3E – monomeric NeonGreen (mNG)

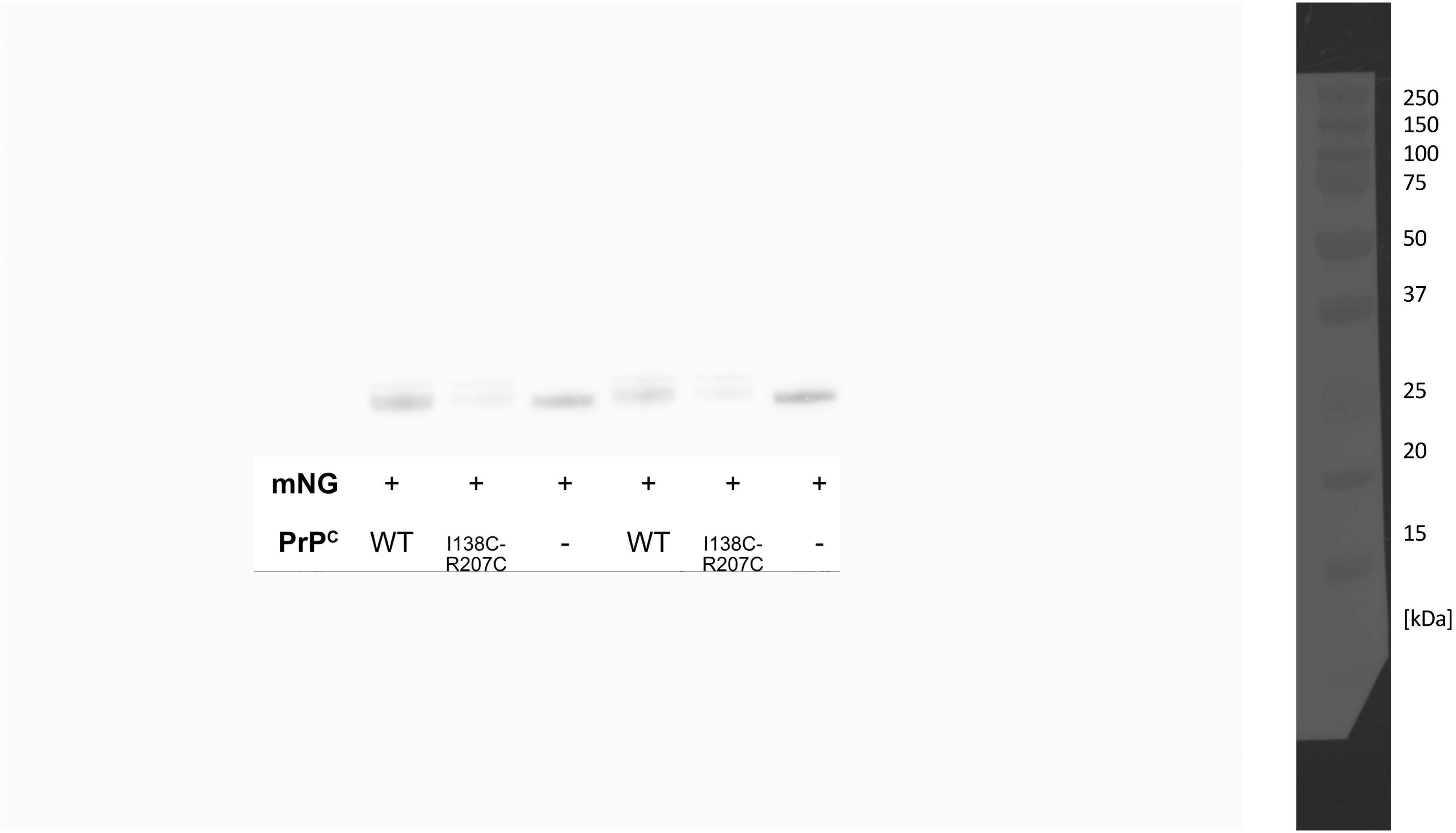

ED Fig 3E – actin

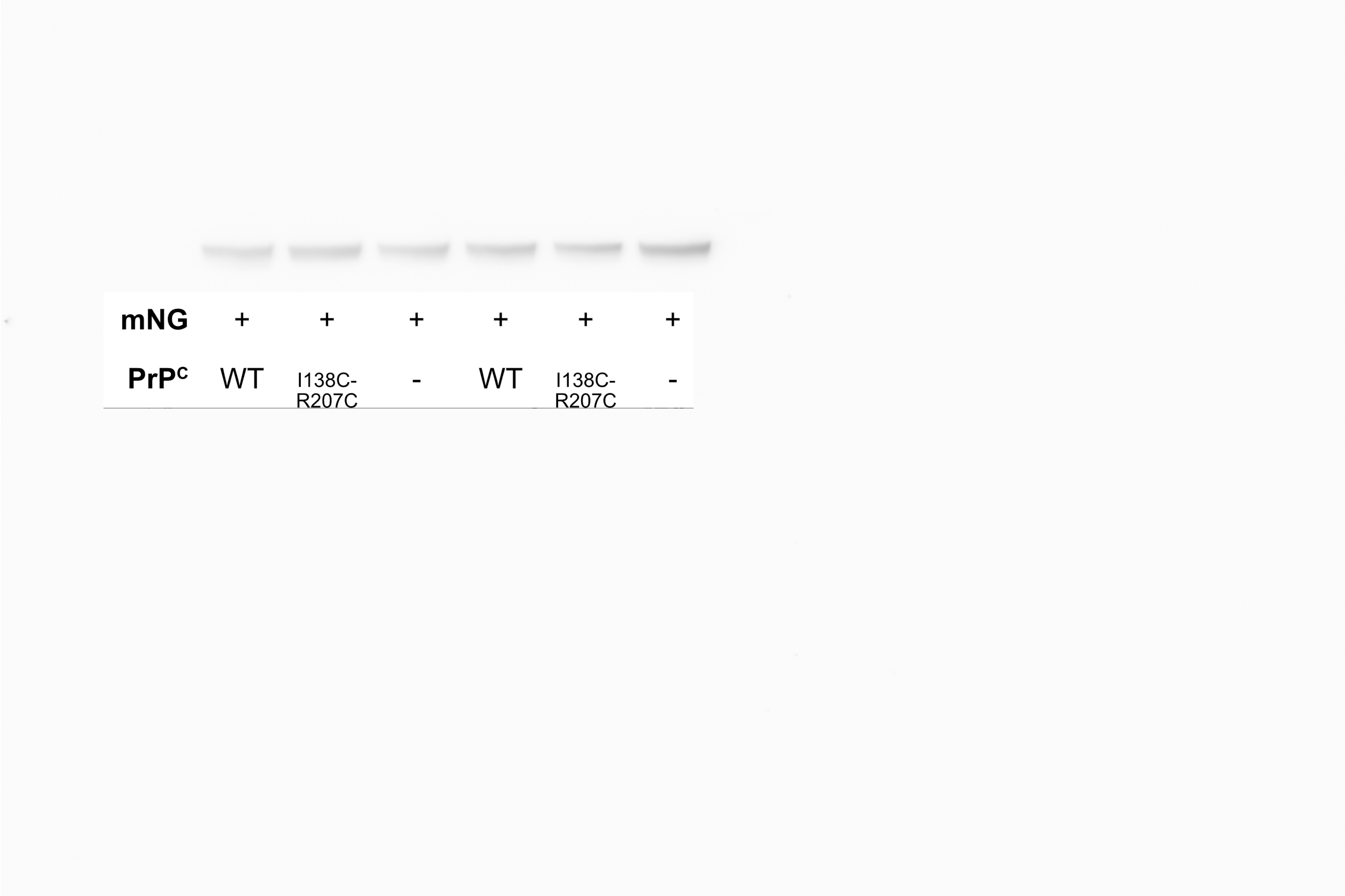

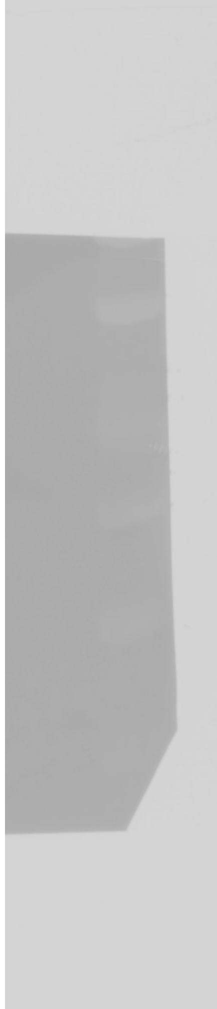

50

37

25

20

15

[kDa]

After mNG exposure, the upper part of the membrane was separated and probed with another antibody
